# Injectable, Antioxidative, and Tissue‐Adhesive Nanocomposite Hydrogel as a Potential Treatment for Inner Retina Injuries

**DOI:** 10.1002/advs.202308635

**Published:** 2024-01-17

**Authors:** Yi‐Chen Liu, Yi‐Ke Lin, Yu‐Ting Lin, Che‐Wei Lin, Guan‐Yu Lan, Yu‐Chia Su, Fung‐Rong Hu, Kai‐Hsiang Chang, Vincent Chen, Yi‐Cheun Yeh, Ta‐Ching Chen, Jiashing Yu

**Affiliations:** ^1^ Department of Chemical Engineering National Taiwan University Taipei 10617 Taiwan; ^2^ Department of Ophthalmology College of Medicine National Taiwan University Taipei 100233 Taiwan; ^3^ Institute of Polymer Science and Engineering National Taiwan University Taipei 10617 Taiwan; ^4^ Department of Ophthalmology National Taiwan University Hospital Taipei 100225 Taiwan; ^5^ Center of Frontier Medicine National Taiwan University Hospital Taipei 100225 Taiwan

**Keywords:** antioxidant hydrogel, gelatin, injectable hydrogel, nanoparticles, optic nerve, photo‐crosslinkable, retinal tissue

## Abstract

Reactive oxygen species (ROS) have been recognized as prevalent contributors to the development of inner retinal injuries including optic neuropathies such as glaucoma, non‐arteritic anterior ischemic optic neuropathy, traumatic optic neuropathy, and Leber hereditary optic neuropathy, among others. This underscores the pivotal significance of oxidative stress in the damage inflicted upon retinal tissue. To combat ROS‐related challenges, this study focuses on creating an injectable and tissue‐adhesive hydrogel with tailored antioxidant properties for retinal applications. GelCA, a gelatin‐modified hydrogel with photo‐crosslinkable and injectable properties, is developed. To enhance its antioxidant capabilities, curcumin‐loaded polydopamine nanoparticles (Cur@PDA NPs) are incorporated into the GelCA matrix, resulting in a multifunctional nanocomposite hydrogel referred to as Cur@PDA@GelCA. This hydrogel exhibits excellent biocompatibility in both in vitro and in vivo assessments, along with enhanced tissue adhesion facilitated by NPs in an in vivo model. Importantly, Cur@PDA@GelCA demonstrates the potential to mitigate oxidative stress when administered via intravitreal injection in retinal injury models such as the optic nerve crush model. These findings underscore its promise in advancing retinal tissue engineering and providing an innovative strategy for acute neuroprotection in the context of inner retinal injuries.

## Graphical abstract

This study addresses inner retinal injuries, emphasizing the role of reactive oxygen species (ROS). GelCA, a photo‐crosslinkable and injectable hydrogel synthesized by grafting cinnamic acid onto gelatin is developed. Incorporating curcumin‐loaded polydopamine nanoparticles results in Cur@PDA@GelCA, a multifunctional nanocomposite hydrogel. This innovative hydrogel demonstrates excellent biocompatibility, enhanced tissue adhesion, and the potential to mitigate oxidative stress in retinal injury models.

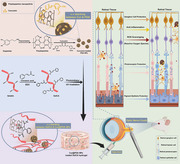

## 1 Introduction

Oxidative damage is implicated in various pathological conditions including tissue degeneration, inflammation, and aging. In the field of tissue engineering, researchers strive to develop innovative strategies to regenerate or repair damaged tissues. This involves the utilization of biomaterials, cells, and biochemical molecules to prevent further damage to the tissues.

Retinal tissue is a highly specialized and crucial component of the visual system, playing a vital role in converting light signals into electrical impulses transmitted to the brain for visual perception. This delicate tissue comprises various types of retinal neurons including retinal ganglion cells (RGC), photoreceptors, bipolar cells, and retinal pigment epithelium (RPE) cells. Due to its high oxygen demand, it is particularly susceptible to oxidative damage, especially damage caused by reactive oxygen species (ROS).^[^ ^1^, ^2^, ^3^ ^]^ This ROS‐induced damage in retinal tissue has been associated with the development of several ocular diseases, encompassing outer retinal injuries, like age‐related macular degeneration,^[^ ^4^, ^5^ ^]^ diabetic retinopathy,^[^ ^6^ ^]^ retinal artery occlusion, and retinal vein occlusion,^[^ ^7^, ^8^ ^]^ as well as inner retinal injuries like glaucoma,^[^ ^9^ ^]^ non‐arteritic anterior ischemic optic neuropathy,^[^ ^10^ ^]^ traumatic optic neuropathy,^[^ ^11^ ^]^ Leber hereditary optic neuropathy,^[^ ^12^ ^]^ and more. If left untreated, these conditions can ultimately lead to ocular neurodegeneration and permanent vision loss.

To combat the various damages associated with retinal diseases, extensive research has focused on the use of therapeutic molecules with effective properties, localized concentration, and long‐lasting effects. For example, the current treatment approach for glaucoma, the most common cause of inner retina and optic nerve degeneration, primarily relies on topical ocular pressure‐lowering medications. These medications provide indirect neuroprotection but often suffer from limited bioavailability when administered through ocular drops.^[^ ^13^, ^14^ ^]^ Intravitreal anti‐VEGF injections are widely used for retinal vascular diseases, but they require repeated injections without a sustained‐release carrier, which can be burdensome in clinical practice.^[^ ^15^, ^16^, ^17^, ^18^, ^19^ ^]^ To address these challenges, a slowly‐releasing delivery system has been applied in the use of intraocular dexamethasone.^[^ ^9^, ^20^, ^21^, ^22^, ^23^ ^]^ However, it may float in the vitreous humor, resulting in unstable and unpredictable local concentrations. To enhance the drug delivery system, an injectable and tissue‐adhesive hydrogel platform may be particularly beneficial, especially for inner retina injuries, given the proximity of the neuronal body of RGC to the inner retinal surface. In the context of countering retinal ROS damage specifically, the development of an antioxidant‐rich hydrogel that can be administered via intravitreal injection holds great promise.

Hydrogels, specifically those derived from natural polymers, such as alginate, gelatin, and collagen,^[^ ^24^, ^25^ ^]^ have demonstrated superior biocompatibility, biodegradability, and mechanical properties compared to synthetic hydrogels. In addition to their ability to mimic the extracellular matrix (ECM), hydrogels can serve as versatile drug delivery platforms due to their porous structure.^[^ ^26^, ^27^ ^]^ Furthermore, the mechanical properties of hydrogels can be tailored and their chemical structures can be modified to enhance their clinical utility.^[^ ^28^, ^29^, ^30^ ^]^ Through these advantages, hydrogels hold great potential for a wide range of applications in the field of regenerative medicine and tissue engineering.

Gelatin, a partially hydrolyzed single‐chain protein derived from collagen, has found extensive use as a substrate for cell growth and as a scaffold material in tissue engineering applications. Its versatility has made it a popular choice for wound healing, tissue repair, and drug delivery systems. Various strategies have been employed in the past to functionalize gelatin including covalent attachment of chemical groups^[^ ^31^, ^32^ ^]^ and photo‐crosslinking techniques.^[^ ^33^, ^34^, ^35^ ^]^ In our study, however, we have taken a unique approach by grafting cinnamic acid onto the gelatin sequence, thereby enabling the formation of a novel photo‐crosslinkable hydrogel, referred to as GelCA. This modification not only introduces new properties to the gelatin‐based hydrogel but also enhances its suitability for applications in tissue engineering and regenerative medicine.

**Figure 1:**
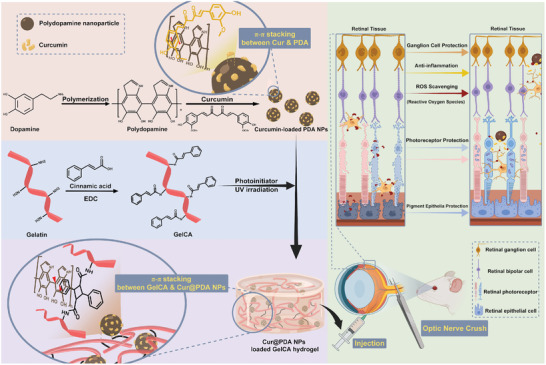
Schematic illustration of the synthesis of Cur@PDA@GelCA hydrogel and its therapeutic mechanism in in vivo retinal optic nerve injury model.

Cinnamic acid, classified as an organic compound of the phenylpropane family, is naturally occurring in numerous plant species. The presence of a carboxyl group within its molecular structure allows for chemical crosslinking with the lysine residues of gelatin,^[^ ^36^ ^]^ thereby yielding a modified gelatin variant termed GelCA. There have been previous studies related to GelCA materials, such as investigating the influence of the duration of UV irradiation on the cross‐linking of gelatin nanofibers containing cinnamic acid, which subsequently affects the swelling behavior of GelCA materials.^[^ ^37^ ^]^ Additionally, a previous study demonstrated the use of microgel particles composed of cinnamic acid‐conjugated gelatin (CA‐GelB) and cinnamic acid‐conjugated Pluronic F127 (CA‐Plur) to control the sustained release of Doxorubicin through electrostatic adsorption.^[^ ^38^ ^]^ Notably, the alkene (C═C) functional group present in cinnamic acid can undergo photo dimerization when exposed to UV irradiation^[^ ^39^, ^40^ ^]^ and in the presence of appropriate photoinitiators, as illustrated in **Figure** **1**. Furthermore, the inclusion of the phenyl functional group within cinnamic acid enhances the affinity of GelCA for adsorbing lipophilic molecules.^[^ ^41^, ^42^ ^]^ Consequently, GelCA exhibits considerable potential for a wide range of applications, notably in the development of efficient drug delivery systems. Our research findings additionally demonstrate that GelCA displays exceptional viscoelastic properties, rendering it suitable as an injectable carrier for clinical applications.

Polydopamine nanoparticles (PDA NPs) are a type of nanoparticle that is formed by the self‐polymerization of dopamine, a natural catecholamine. The formation of PDA NPs is a simple, one‐step process that is carried out by exposing dopamine to an oxidizing agent in an aqueous environment. The characteristics of PDA nanoparticles are their biocompatibility and biodegradability, coupled with their strong π–π stacking interactions and hydrogen bonding abilities,^[^ ^43^ ^]^ making them useful for biomedical applications such as drug delivery,^[^ ^44^ ^]^ tissue engineering, and biosensors. For example, studies have used PDA nanoparticles as carriers for drugs such as doxorubicin,^[^ ^45^ ^]^ oxaliplatin,^[^ ^46^ ^]^ and brimonidine.^[^ ^47^ ^]^ Additionally, PDA nanoparticles have been shown to have antioxidant^[^ ^48^, ^49^ ^]^ and anti‐inflammatory properties,^[^ ^50^, ^51^ ^]^ making them useful for treating diseases related to oxidative stress and inflammation.

Curcumin, a natural compound derived from the spice turmeric, is classified as a phenolic pigment due to its distinctive chemical structure. Extensive research has demonstrated that curcumin possesses notable anti‐inflammatory, antioxidant, and antibacterial properties,^[^ ^52^, ^53^ ^]^ positioning it as a promising natural remedy for various health conditions. One key advantage of curcumin is its relatively low toxicity profile, rendering it unlikely to induce severe side effects. However, despite its numerous benefits, curcumin encounters certain limitations. One of these limitations is its poor solubility and limited distribution within the body, particularly at physiological pH.^[^ ^54^ ^]^ Consequently, effective absorption and utilization of curcumin in vivo can be challenging. Additionally, curcumin undergoes rapid metabolism, leading to swift breakdown and elimination, thereby further curtailing its therapeutic effectiveness. In response, researchers are actively exploring diverse strategies to enhance its bioavailability. Notably, a promising approach involves the adsorption of curcumin onto polydopamine nanoparticles (Cur@PDA NPs) through π–π stacking interactions.^[^ ^52^, ^55^ ^]^ This adsorption process enables curcumin to well distribute in the aqueous system, thereby enhancing its bioavailability and augmenting its therapeutic efficacy.

In this study, we have successfully developed a GelCA hydrogel incorporated with curcumin‐loaded polydopamine nanoparticles (Cur@PDA@GelCA), which exhibits excellent injectability, tissue adhesive properties, and antioxidant properties. This innovative hydrogel holds great promise as a prospective clinical material for addressing retinal ROS‐related disorders. By leveraging the unique characteristics of the GelCA platform and the antioxidative effects of curcumin, our study contributes to the advancement of therapeutic approaches for inner retinal tissue damage. Further investigations and clinical trials are warranted to fully explore the potential of Cur@PDA@GelCA hydrogel in the field of ophthalmology and tissue engineering.

## 2 Results and Discussion

### 2.1 Characterizations of Cinnamic Acid‐Modified Gelatin (GelCA)

**Figure 2:**
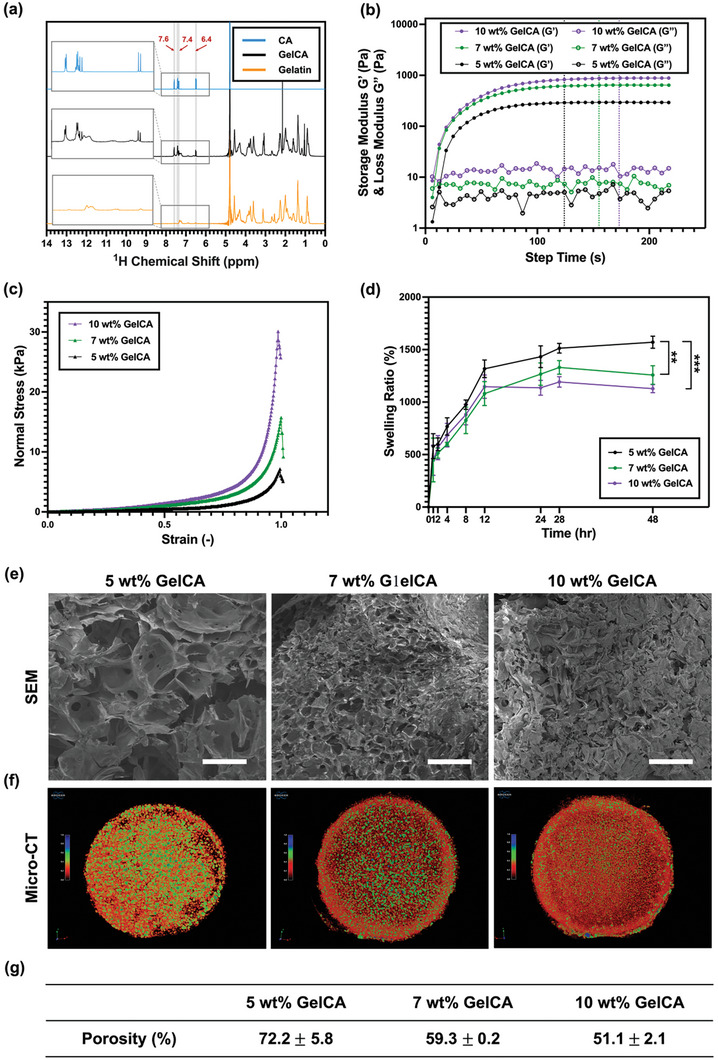
Characterizations of modified gelatin (GelCA) and the physical properties and morphology of GelCA hydrogel. a) ^1^H NMR spectra of cinnamic acid, gelatin, and GelCA. b) Time‐dependent rheological properties of GelCA solution under 25 mW cm^−^^2^ UV irradiation. Solid circles represent the storage modulus of the hydrogel's rheological behavior, while hollow circles represent the loss modulus. Rheological measurements were conducted using an 8 mm‐diameter plate geometry set at a 0° angle at room temperature. An oscillatory time sweep was performed at an angular frequency of 10 rad s^−1^ with a strain of 1%. c) The compression properties of the GelCA hydrogel were evaluated using a texture analyzer. d) The swelling ratio of GelCA hydrogel (*n* = 3). e) SEM images of GelCA hydrogel (scale bars: 300 µm). f) Micro‐CT image of GelCA hydrogel. g) The table presents the porosity of GelCA hydrogel at various concentrations, determined using a mercury porosimeter.

GelCA was synthesized by covalently bonding cinnamic acid, a natural compound, to gelatin through the formation of amide bond bonds between the carboxyl group on cinnamic acid and the amino group on gelatin, as depicted in Figure 1. In this study, the synthesis of GelCA was confirmed through ^1^H nuclear magnetic resonance (NMR) analysis. The NMR spectra, shown in **Figure** **2**a, revealed distinct chemical shifts at 7.4 and 7.5 ppm for arene C═C and 6.4 ppm for alkene C═C chemical structures of pure cinnamic acid.^[^ ^37^, ^56^ ^]^ These peaks served as evidence that cinnamic acid was successfully grafted onto the gelatin. Comparing the spectra of gelatin and GelCA confirmed the desired outcome, as GelCA exhibited additional peaks at around 6.4, 7.4, and 7.5 ppm compared to gelatin. These additional peaks indicated the presence of alkene C═C and arene C═C functional groups from cinnamic acid in the modified gelatin (GelCA).

As indicated in Figure S1, Supporting Information, it was anticipated that distinctive peaks corresponding to the functional groups of cinnamic acid would appear in the Fourier‐transform infrared (FTIR) spectrum of modified GelCA, specifically around 1680, 1630, 1580, and 1500 cm^−1^ (representing C═O stretching, alkene C═C stretching, and arene C═C stretching). However, no discernible difference was observed in the spectrum of GelCA compared to pure gelatin. This can be attributed to the fact that GelCA was produced by grafting cinnamic acid onto the lysine amino groups present in gelatin. Commercial gelatin (type B) typically contains a relatively low lysine content, comprising only about 2.76% of the total amino acid sequence.^[^ ^57^ ^]^ Consequently, distinguishing the dissimilarity between modified gelatin and GelCA in low‐resolution FTIR spectra became challenging.

The modification of GelCA followed a high degree of substitution method based on a previous protocol.^[^ ^37^ ^]^ The molar ratio of gelatin to cinnamic acid to 1‐ethyl‐(3‐dimethylaminopropyl) carbodiimide (EDC) was set as 1:9:90. To analyze the degree of substitution of GelCA, we employed the 2,4,6‐trinitrobenzenesulfonic acid (TNBS) assay. Unmodified gelatin, containing lysine residues with primary amines, reacted with TNBS, resulting in an absorption peak at 353 nm. Concurrently, the bicinchoninic acid (BCA) assay was used to normalize the protein concentration. By dividing the concentration of primary amines in the GelCA and gelatin solutions and considering the original concentration of the protein, the degree of substitution of GelCA was calculated to be 91%.

Additionally, GelCA can undergo photocrosslinking to form GelCA hydrogels through the dimerization of alkene functional groups on cinnamic acid, facilitated by a photoinitiator and UV irradiation. To investigate the physical properties of GelCA hydrogels formed through photocrosslinking, rheological properties, compressive strength, and water absorption tests were conducted on GelCA hydrogels with concentrations of 5, 7, and 10 wt%. As depicted in Figure 2b, the rheological results were obtained from 5, 7, and 10 wt% GelCA solutions under the influence of a photoinitiator and 25 mW cm^−2^ UV irradiation. The results demonstrated that all three concentrations of GelCA solutions exhibited a crossover point between the storage modulus and the loss modulus during the initial stage of UV light exposure (≈10 s), indicating the transition of GelCA solutions from a viscous material to a viscoelastic material under UV irradiation. Furthermore, with continued exposure to UV irradiation, the storage modulus of the 5 wt% GelCA solution reached a plateau (saturated curing point) at ≈124 s, while the 7 and 10 wt% GelCA solutions at ≈155 and 173 s, respectively. These observations suggest that at these time points, the GelCA within the solution reached a saturated curing point for dimerization, resulting in the formation of structurally stable GelCA hydrogels through photocrosslinking.

In addition, compressive stress was also employed to investigate the mechanical properties of 5, 7, and 10 wt% GelCA hydrogels. Figure 2c illustrates the strain–stress trend curves of the GelCA hydrogels with three concentrations after complete photopolymerization. All three concentrations of GelCA hydrogels exhibited stable deformation under compressive stress, and structural failure occurred only when the strain reached ≈99%, indicating excellent deformability of GelCA hydrogels. Furthermore, Young's modulus of the GelCA hydrogels at specific strain rates was examined. The Young's modulus of 5 wt% hydrogels was found to be 1.26  ±  0.08 kPa, while 7 and 10 wt% hydrogels had values of 3.26  ±  0.06 and 5.27  ±  0.13 kPa, respectively. This demonstrates that, under the same applied stress, GelCA hydrogels with lower concentrations exhibit greater deformation.

In order to gain a more comprehensive understanding of the characteristics of GelCA hydrogel, we also conducted swelling tests on three different concentrations of GelCA hydrogel. Photocrosslinked hydrogels possess a 3D porous structure, which enables them to adsorb and retain a large amount of water. From the perspective of the liquid uptake ratio (Figure 2d), the swelling rate of the three concentrations of GelCA hydrogel tends to reach a constant value after rehydration for ≈24 h following lyophilization. After 48 h, the swelling ratio of the 5 wt% GelCA hydrogel attained the highest value among the three groups, ≈1570%, indicating that this concentration of hydrogel has the highest liquid uptake capacity. This is attributed to the lower density of photocrosslinking points, resulting in lower hydrogel expansion constraint and higher porosity. Therefore, under similar osmotic pressures, the 5 wt% GelCA hydrogel exhibits the highest swelling ratio.

A scanning electron microscope (SEM) was also used to observe the detailed structure of GelCA hydrogel at the micrometer scale. As shown in Figure 2e, all three concentrations of GelCA hydrogel exhibit a porous 3D morphology. As the concentration increases, the pore size in the hydrogel decreases, and at a concentration of 10 wt%, it becomes difficult to observe regular micrometer‐scale pores. The SEM images correspond to the results obtained from rheological tests and compression tests. As the concentration of the hydrogel increases, the number of photocrosslinking points also increases, resulting in a longer time required to reach the saturation point for dimerization and a longer time to achieve a stable value for the storage modulus. The SEM images also reveal that hydrogels with lower concentrations have larger pores. Therefore, during compression testing, these hydrogels exhibit a lower Young's modulus due to the lower overall support provided by the hydrogel scaffold.

In order to investigate the overall pore distribution and porosity of GelCA hydrogels at different concentrations, the scaffold structure of GelCA hydrogels was scanned using micro‐computed tomography (Micro‐CT), as shown in Figure 2f. Represented by different colors in the image were pores of different sizes. Indicated by a higher value on the color bar was a larger pore size. However, the value itself did not represent the actual size of the pore. The Micro‐CT images revealed that the pore structure of GelCA hydrogel scaffolds was uniform across different concentrations, with the largest pore size observed in the 5 wt% GelCA, followed by 7 wt% GelCA, and finally 10% GelCA. To further determine the porosity of GelCA hydrogels, mercury porosimetry was employed and the results are shown in Figure 2g. The porosity of the 5 wt% GelCA hydrogel was found to be the highest at ≈72.2%, while the porosities of the 7 and 10 wt% GelCA hydrogels were measured to be 59.3% and 51.1%, respectively. These findings align with the results of the swelling ratio, as the 5 wt% GelCA hydrogel exhibited the highest porosity, providing more space for water uptake and consequently resulting in the highest swelling ratio. However, it is worth noting that when the hydrogel dries and undergoes deformation under pressure, the reorganization and compaction of the hydrogel may cause the collapse of certain smaller pores, thereby impeding the further intrusion of mercury. This can potentially lead to an underestimation of porosity values, resulting in underestimated porosity values when testing the hydrogel using a mercury porosimeter.

### 2.2 Characterizations of PDA and Cur@PDA Nanoparticles

**Figure 3:**
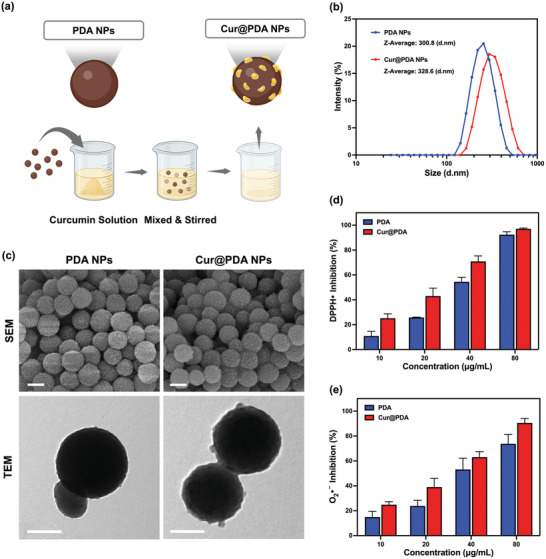
Physical and chemical properties of the nanoparticles. a) Schematic representation of the synthesis of Cur@PDA Nanoparticles. b) Size distribution. c) SEM images (scale bars: 150 nm) and TEM images (scale bars: 150 nm) of PDA, Cur@PDA nanoparticles. d) DPPH• free radical scavenging activity of PDA and Cur@PDA NPs at different concentrations (*n* = 3). e) O_2_^• −^ scavenging activity of PDA and Cur@PDA NPs at different concentrations (*n* = 3).

PDA nanoparticles are self‐polymerized through Michael‐type addition and Schiff base reactions in an alkaline environment.^[^ ^58^, ^59^ ^]^ Additionally, the phenolic groups of PDA enable noncovalent binding, such as hydrogen bonding and π–π stacking, allowing for the adsorption of small molecule drugs. Therefore, we loaded curcumin onto the surface of PDA nanoparticles to synthesize Cur@PDA nanoparticles, as shown in **Figure** **3**a. The Brownian motion of both PDA and Cur@PDA nanoparticles was measured using a Zetasizer, and the temperature and viscosity of the sample were utilized to calculate the electrical double‐layer diameters of the nanoparticles. As depicted in Figure 3b, both PDA and Cur@PDA nanoparticles exhibited Gaussian distributions with a polydispersity index (PDI) of less than 0.3. The size of PDA nanoparticles depends on the pH value, polymerization temperature, and concentration of dopamine hydrochloride.^[^ ^60^ ^]^ To ensure uniformity in size, we maintained the three conditions during the synthesis of PDA nanoparticles. The hydrodynamic diameter of pure PDA nanoparticles was found to have an average value of 300.8 nm. Using a physical adsorption method, we prepared Cur@PDA nanoparticles, and the efficiency of curcumin loading was determined by comparing the amount of curcumin present in the methanol solution before and after drug‐loading fabrication. The absorption peak at 425 nm of curcumin solution was utilized to quantitatively assess the results (Figure S3a, Supporting Information). The results indicated that each milligram of PDA nanoparticles loaded 0.130 milligrams of curcumin. Consequently, the hydrodynamic diameter of the curcumin‐loaded nanoparticles (Cur@PDA) increased to an average of 328.6 nm.

The zeta potential measurements of the nanoparticles were taken, as shown in Figure S3b, Supporting Information, and it was found that the average zeta potential of pure PDA nanoparticles was −29.7  ±  0.6 mV. This is due to the fact that the isoelectric point of PDA nanoparticles is ≈4.6.^[^ ^61^ ^]^ Therefore, in a neutral environment (pH 7), the phenolic groups of PDA nanoparticles undergo protonation, resulting in a stable negative charge on the nanoparticles. On the other hand, the zeta potential of pure curcumin was −15.5  ±  2.7 mV. However, the surface‐loaded curcumin on PDA (Cur@PDA) nanoparticles was found to have a surface potential of −26.7  ±  1.1 mV, as expected. It can be seen that the absolute value of the zeta potential of the nanoparticles slightly decreased after the surface adsorption of curcumin. Certainly, both PDA and Cur@PDA nanoparticles carry stable negative charge in a neutral environment, ensuring good dispersibility and avoiding the common aggregation issues associated with nanoparticles.^[^ ^62^ ^]^

The results of two types of nanoparticles observed using a SEM are shown in Figure 3c. Pure PDA nanoparticles were found to be completely spherical with uniform particle sizes, approximately between 250 and 300 nm. On the other hand, Cur@PDA nanoparticles also had uniform sizes, but no obvious increase in particle size was observed compared to PDA nanoparticles when viewed through SEM. It was observed that the surface of the nanoparticles became rougher and irregular curcumin particles appeared on the surface of the larger particles after being loaded with curcumin. Additionally, gaps between the nanoparticles were also found to be rich in curcumin. The morphology of the two types of nanoparticles was also observed using a transmission electron microscope (TEM), as shown in Figure 3c. The electron transmittance of pure PDA nanoparticles was found to be uniform, resulting in a uniform black spherical shape. In Cur@PDA nanoparticles, a notable distinction arose from the variance in electron transmittance between curcumin and PDA, leading to the discernible presence of curcumin on the exterior of the PDA nanoparticles.

DPPH• free radicals were utilized to test the efficiency of two types of nanoparticles in clearing ROS. As shown in Figure 3d, all sample groups at high concentrations (80 µg mL^−1^) had no absorption value at 517 nm after the reaction, indicating that all samples could reduce free radicals in the solution with this concentration completely. However, the inhibition effect of PDA nanoparticles was observed to have improved as the sample concentration was increased, indicating that the concentration of nanoparticles played a role in removing free radicals. At a high concentration (80 µg mL^−1^) of PDA nanoparticles, the DPPH• inhibition effect was nearly 100%. The performance of Cur@PDA nanoparticles in removing DPPH• was found to be better than that of PDA nanoparticles at the same concentration, and the percentage of DPPH• inhibition in 10 µg mL^−1^ Cur@PDA groups was found to be two times higher than that in PDA nanoparticle groups. The colorimetric results of the DPPH• free radical reaction with nanoparticles are shown in Figure S4a, Supporting Information.

In addition, we verified the scavenging efficiency of PDA and Cur@PDA nanoparticles on various types of ROS using superoxide, O_2_^• −^. Typically, when exposed to 365 nm UV irradiation in the presence of riboflavin, l‐methionine, and nitro blue tetrazolium (NBT), a robust absorbance signal at 560 nm was observed, indicating the presence of high levels of O_2_^• −^ in the solution. However, upon the addition of PDA and Cur@PDA nanoparticles to the solution, both were effective in reducing the O_2_^• −^ levels, leading to a decrease in the 560 nm absorbance signal. Specifically, statistical results are shown in Figure 3e, while colorimetric results can be found in Figure S5, Supporting Information, illustrating that as the concentration of nanoparticles increased, the scavenging efficiency of O_2_^• −^ also improved. Nevertheless, when PDA nanoparticles were present at a concentration of 80 µg mL^−1^, they effectively removed around 70% of O_2_^• −^ from the solution. In contrast, with the same concentration of Cur@PDA nanoparticles, ≈90% of O_2_^• −^ was removed from the solution. This demonstrated the superior capacity of Cur@PDA nanoparticles for O_2_^• −^ removal compared to PDA nanoparticles. These findings certified that Cur@PDA outperformed PDA nanoparticles in terms of both DPPH• free radicals and O_2_^• −^ scavenging efficiency, regardless of the nanoparticle concentrations.

### 2.3 Characterizations of Nanocomposite GelCA Hydrogels

Considering the characteristics observed in testing different concentrations of GelCA hydrogel, the 5 wt% hydrogel exhibited faster complete photocuring, greater susceptibility to compression‐induced deformation, and a larger swelling ratio. In subsequent in vivo optic nerve model (ONC) experiments, the hydrogels are administered to the retina through intravitreal injection. Given that ≈98–99% of the components in the vitreous tissue are composed of water,^[^ ^63^ ^]^ hydrogels with a high swelling ratio ensure that they are fully saturated with a higher percentage of water prior to injection. This allows hydrogels to more closely align with the natural water content of the vitreous tissue when introduced into the retinal tissue. Therefore, for the subsequent experiments, the 5 wt% GelCA hydrogel was chosen for loading PDA and Cur@PDA nanoparticles.

**Figure 4:**
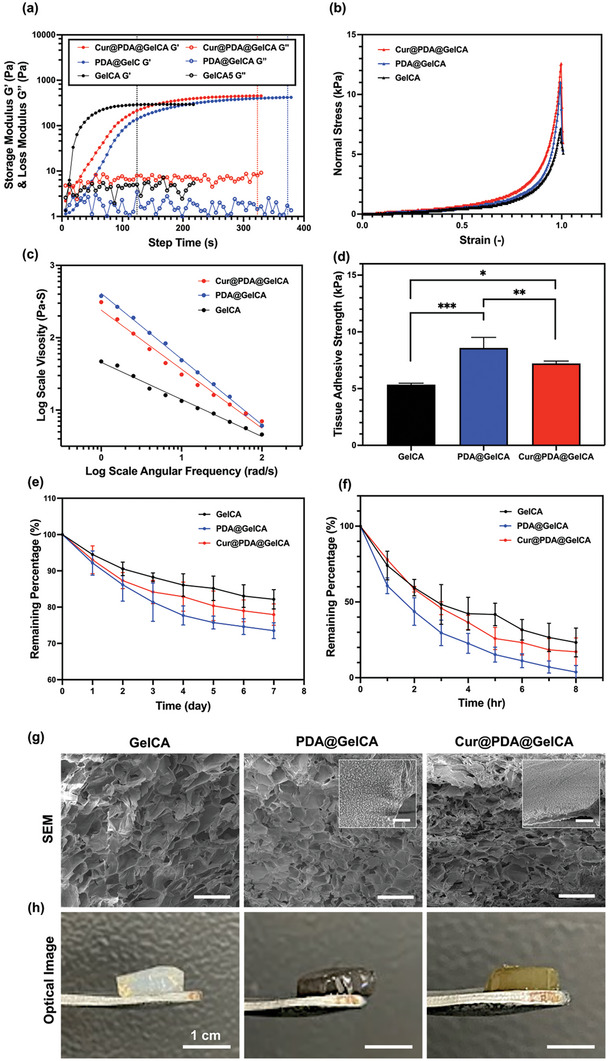
Physical and chemical properties of GelCA, PDA@GelCA, and Cur@PDA@GelCA hydrogels. a) Time‐dependent rheological properties of GelCA, PDA@GelCA, and Cur@PDA@GelCA precursor solution under 25 mW cm^−^^2^ UV irradiation. Solid circles represent the storage modulus of the hydrogel's rheological behavior, while hollow circles represent the loss modulus. Rheological measurements were conducted using an 8 mm‐diameter plate geometry (0° angle) at room temperature. An oscillatory time sweep was performed at an angular frequency of 10 rad s^−1^ with a strain of 1%. b) Compression properties of GelCA, PDA@GelCA, and Cur@PDA@GelCA hydrogels. c) Shear‐thinning test by continuous flow sweep of the GelCA, PDA@GelCA, and Cur@PDA@GelCA hydrogels. The shear rate was set between 1 and 100 s^−1^ with 1% strain using a 20 mm‐diameter plate geometry (0° angle) at 37 °C. d) Tissue adhesive strength test of GelCA, PDA@GelCA, and Cur@PDA@GelCA hydrogels through the lap‐shear adhesion measurement, the data are presented as mean ± SD (*n* = 3). e) The long‐term dissolution rate of GelCA, PDA@GelCA, and Cur@PDA@GelCA hydrogels in DMEM/HG medium, the data are presented as mean ± SD (*n* = 3). f) The short‐term degradation rate of GelCA, PDA@GelCA, and Cur@PDA@GelCAhydrogels in collagenase solution, the data are presented as mean ± SD (*n* = 3). g) SEM images illustrating the cross‐sectional morphology of the GelCA, PDA@GelCA, and Cur@PDA@GelCA hydrogels (short scale bars: 10 µm, long scale bars: 300 µm). h) The visual appearance of 300 µL GelCA, PDA@GelCA, Cur@PDA@GelCA hydrogels.

In order to examine the mechanical characteristics of the GelCA hydrogel incorporating PDA and Cur@PDA nanoparticles (PDA@GelCA and Cur@PDA@GelCA), rheological tests were performed on PDA@GelCA, Cur@PDA@GelCA solutions, and pure GelCA solution under 25 mW cm^−2^ UV irradiation (365 nm), as shown in **Figure** **4**a. With the incorporation of PDA and Cur@PDA nanoparticles into the 5% GelCA nanocomposites, the cross‐over points between the loss modulus and storage modulus experience a delay. Specifically, the cross‐over point for PDA@GelCA is extended to ≈31 s, whereas the Cur@PDA@GelCA cross‐over point is observed at around 28 s. Moreover, the results revealed that the addition of PDA and Cur@PDA nanoparticles increased the time required for GelCA hydrogel to reach complete photodimerization. The time increased from 124 (GelCA group) to 372 (PDA@GelCA group) and 323 s (Cur@PDA@GelCA group), indicating spatial hindrance of GelCA dimerization due to the presence of nanoparticles. Among the two groups, the PDA nanoparticles had a greater impact on photodimerization compared to the equal‐weight Cur@PDA nanoparticles. However, both groups achieved stable storage modulus within a few minutes of UV irradiation, demonstrating that the presence of PDA and Cur@PDA nanoparticles did not affect the stability of complete gelation. Next, the storage modulus of the three groups was compared after complete photodimerization. The GelCA group had a final modulus of ≈291.1  ±  1.9 Pa, while the PDA@GelCA and Cur@PDA@GelCA groups had moduli of ≈418.8  ±  0.8 and 451.4  ±  1.4 Pa, respectively. This result indicated a significant increase in the rigidity of the GelCA hydrogel after the addition of nanoparticles, with the Cur@PDA@GelCA group showing a greater enhancement.

Additionally, compression tests were performed to assess the mechanical properties of the three hydrogels, as shown in Figure 4b. It was observed that the Young's Modulus (15–20% Strain) of PDA@GelCA and Cur@PDA@GelCA was greater than that of pure GelCA, with values of 1.87  ±  0.11 (PDA@GelCA), 2.09  ±  0.08 (Cur@PDA@GelCA), and 1.26  ±  0.08 kPa (GelCA), respectively. The previous results showed that porcine ocular tissues exhibited an average compressive modulus of 10.5 ± 2.67 kPa within a 0–10% strain range, whereas murine ocular tissues (wildtype) displayed compressive moduli ranging from 15.2 ± 4.08 to 41.9 ± 11.99 kPa within the same strain range, with variations based on the source of retina samples.^[^ ^64^ ^]^ However, the three hydrogels in our study, GelCA, PDA@GelCA, and Cur@PDA@GelCA, demonstrated compressive moduli of 0.54 ± 0.02, 1.21 ± 0.03, and 0.52 ± 0.02 kPa, respectively, within the 0–10% strain range, as shown in Figure 4b. These results indicate that the compressive moduli of the three hydrogels are lower than those of real murine and porcine ocular tissues, signifying that the hydrogels are softer than the tissues. This property makes them less likely to cause damage to the retina when injected into the ocular environment.

To investigate the syringe injection properties of hydrogels, we assessed the viscosity of three types of hydrogels at varying shear rates (1–100 s^−1^). As the shear rate increased for all hydrogel formulations, a decrease in viscosity was observed (Figure 4c). This observation indicates that GelCA hydrogel can transition from its initial random structure to an oriented arrangement at higher shear frequencies, a behavior also confirmed for the nanocomposite hydrogels containing PDA and Cur@PDA NPs. Consequently, this study validates the non‐Newtonian behavior of the three hydrogels, making them suitable candidates for syringe injection applications. Applying the power‐law equation, we determined the flow index (*n* value) for GelCA, PDA@GelCA, and Cur@PDA@GelCA hydrogels to be 0.48 ± 0.07, 0.12 ± 0.04, and 0.18 ± 0.03, respectively. This analysis demonstrates that the addition of nanoparticles leads to more pronounced shear‐thinning behavior in these hydrogels. Furthermore, the evaluation of the consistency index (K value) reveals values of 47.7 ± 13 for GelCA, 413 ± 15 for PDA@GelCA, and 242 ± 11 for Cur@PDA@GelCA hydrogels. These results indicate that GelCA exhibits relatively low viscosity at the same shear rate, which increases upon the addition of nanoparticles. At low shear rates, PDA@GelCA and Cur@PDA@GelCA exhibit higher viscosities, but under the influence of higher shear rates, their viscosity tends to approach that of pure GelCA hydrogel. To assess the healing properties of three different hydrogels after the removal of shear forces. As depicted in Figure S8a, Supporting Information, all three hydrogels consistently exhibited a return to a storage modulus approximately equivalent to their original state after undergoing multiple cycles of low strain (0.1%) and high strain (1000%). This phenomenon is presumed to be a result of the disruption of dimerization between GelCA macromers under high‐strain conditions. However, when returning to low strain, the aromatic rings on Cinnamic acid are believed to facilitate π–π stacking interactions. This hypothesis is supported by previous research indicating that aromatic interactions contribute to the reconstruction of hydrogel networks following damage.^[^ ^65^, ^66^ ^]^ Additionally, the inclusion of PDA and Cur@PDA NPs in the hydrogels enhances their self‐healing capabilities at 37 °C during rheological tests. This enhancement is attributed to the rich presence of π–π stacking and hydrogen bonding within these hydrogel networks.

To further assess the in vivo tissue utility of the hydrogels, we conducted tissue adhesion experiments and investigated the curcumin release efficiency from Cur@PDA@GelCA hydrogel. Initially, we employed the Porcine Skin lap‐shear test method to examine the influence of these hydrogels on tissue adhesion strength. Figure 4d reveals that the PDA@GelCA hydrogel exhibits the highest shear strength at 8.58 ± 0.809 kPa, followed by PDA@Cur@GelCA 7.21 ± 0.182 kPa, with the weakest adhesion observed in the pure GelCA hydrogel (5.35 ± 0.108 kPa). These results indicate that the original GelCA hydrogel possesses relatively lower tissue adhesiveness. However, upon the addition of PDA and Cur@PDA NPs, these functional groups exhibit a strong affinity for nucleophilic agents, such as amines and thiols present on the tissue surface,^[^ ^67^, ^68^ ^]^ leading to an enhancement in tissue adhesion. We also conducted curcumin release experiments to investigate the curcumin release behavior of the Cur@PDA@GelCA hydrogel. As shown in Figure S8b, Supporting Information, the hydrogel demonstrated stable curcumin release in phosphate‐buffered saline (PBS) at 37 °C. The cumulative amount of curcumin released from the hydrogel increases with time, reaching 162 ± 18.8 µg at 168 h (7 days). This accounts for around 70.5 ± 10% of the initial curcumin loading in the hydrogel, which was 230 µg. Furthermore, long‐term dissolution tests in pure medium and short‐term degradation tests with collagenase were performed to examine the stability of the three hydrogel groups in a biological environment, as depicted in Figure 4e,f. In the long‐term dissolution tests, it was observed that over a period of 7 days in the medium environment, the three hydrogel groups gradually dissolved, releasing substances that were not covalently crosslinked within the GelCA structure, such as photoinitiators, uncrosslinked GelCA macromers, and nanoparticles. On the seventh day, the GelCA hydrogel exhibited the highest remaining percentage, while the PDA@GelCA hydrogel had the lowest remaining percentage. This can be attributed to the dissolution of a few nanoparticles from the GelCA hydrogel structure and the impact of incorporated PDA nanoparticles on the crosslinking of GelCA itself, as supported by the results in Figure 4a, which demonstrated an increased saturation time for crosslinking upon nanoparticle addition. However, overall, all three hydrogel groups retained more than 70% of their substances in the medium environment after 7 days. Regarding the short‐term degradation tests, the collagenase type 1 enzyme was utilized to digest the GelCA structure. The results indicated that the presence of nanoparticles accelerated the hydrolysis rate of the GelCA hydrogel. By the eighth hour, the PDA@GelCA group had been completely hydrolyzed, while the GelCA and Cur@PDA@GelCA groups still retained ≈20% of their structure. This finding aligns with the results in Figure 4a, where the presence of nanoparticles reduced the available crosslinking sites in GelCA, particularly in the case of PDA nanoparticles, thereby hastening the digestion of the overall hydrogel structure by collagenase type 1 enzyme.

Figure 4g depicts the microstructure of the three hydrogels, where PDA@GelCA and Cur@PDA@GelCA exhibited the same porous structure as the GelCA hydrogel, with evenly dispersed pores. This indicated that the addition of PDA and Cur@PDA nanoparticles did not affect the microscale architecture of the hydrogel's 3D structure. However, upon closer examination, it was observed that the PDA@GelCA and Cur@PDA@GelCA groups exhibited nanoscale protrusions on the surface of the scaffold. In Figure 4h, the optical images of the three hydrogels are displayed. The pure GelCA hydrogel appears milky white, while the PDA@GelCA hydrogel exhibited a deep brown color due to the presence of PDA nanoparticles. Similarly, the Cur@PDA@GelCA hydrogel appeared yellowish‐brown, corresponding to the color of the Cur@PDA nanoparticles.

The scavenging efficacy of DPPH• free radicals was assessed using three types of hydrogels: GelCA, PDA@GelCA, and Cur@PDA@GelCA. As depicted in Figure S6a,c, Supporting Information, a DPPH• scavenging efficiency of ≈16.7% was exhibited by the GelCA hydrogel. However, the incorporation of NPs was found to significantly enhance the scavenging efficiency of the PDA@GelCA and Cur@PDA@GelCA hydrogels. An increase in NP concentration was associated with increased efficiency; hydrogels containing 40 µg/100 µL hydrogel of NPs were shown to demonstrate scavenging efficiencies of 49.2% and 56.7% for PDA@GelCA and Cur@PDA@GelCA, respectively. When the NP concentration was increased to 80 µg/100 µL hydrogel, these efficiencies escalated to 72.1% and 91.9%. Furthermore, the O2•^−^ scavenging efficiency of these hydrogels was also evaluated. As shown in Figure S6b,d, Supporting Information, the GelCA hydrogels were found to have a minimal scavenging capacity of about 11.6% under the test conditions. However, the integration of PDA and Cur@PDA NPs into the PDA@GelCA and Cur@PDA@GelCA hydrogels was observed to effectively enhance their capacity to reduce O_2_^• −^ levels, as evidenced by the decreased absorbance at 560 nm. Notably, an improvement in O_2_^• −^ scavenging efficiency was observed with increasing concentrations of NPs. Hydrogels with 40 µg NPs/100 µL hydrogel were found to achieve scavenging efficiencies of 51% and 59% for PDA@GelCA and Cur@PDA@GelCA, respectively, and these efficiencies further increased to 68.4% and 89.3% with a concentration of 80 µg NPs/100 µL hydrogel. These results from both ROS scavenging assays clearly demonstrate that the incorporation of NPs markedly boosts the ROS scavenging efficiency of the hydrogels.

### 2.4 In Vitro Antioxidative Performance of Nanoparticle‐Loaded Hydrogel

To evaluate the antioxidant properties of PDA@GelCA and Cur@PDA@GelCA hydrogels in vitro, we employed AmplexTM Red reagent (10‐acetyl‐3,7‐dihydroxyphenoxazine) to assess the oxidative stress of RGC‐5 cells in the presence of hydrogen peroxide. AmplexTM Red is a colorless reagent that under the presence of horseradish peroxidase (HRP), the reaction between the substance and hydrogen peroxide occurs in a 1:1 stoichiometry, leading to the formation of the fluorescent product resorufin.^[^ ^69^, ^70^ ^]^ The results showed that cells in the group without hydrogen peroxide treatment and hydrogel (control group) exhibited minimal resorufin signal. In contrast, cells treated with hydrogen peroxide without the presence of a hydrogel (H_2_O_2_ group) displayed a high‐intensity resorufin signal.

**Figure 5:**
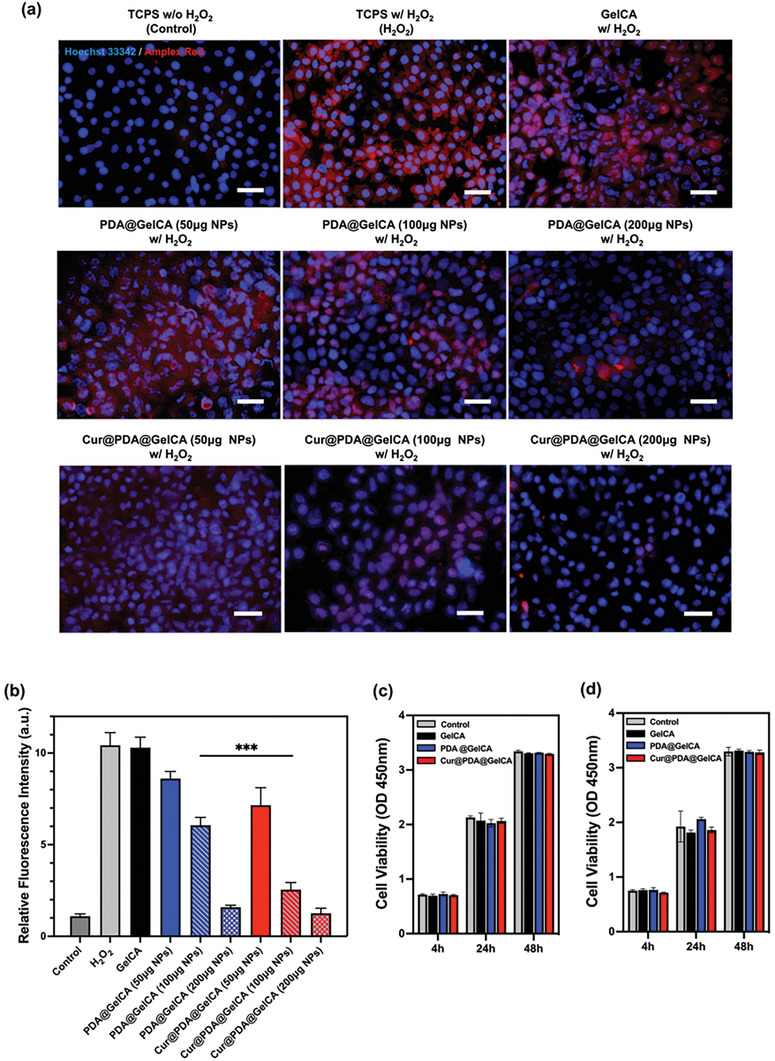
a) Amplex red fluorescence staining of ROS in RGC‐5 and Hoechst 33 342 fluorescence staining of RGC‐5 nucleus (scale bars: 100 µm). b) Quantitative analysis of the relative fluorescence intensity of Amplex red (*n* = 3). c) The absorbance of RGC‐5 viability test by CCK‐8 assay (*n* = 3). d) The absorbance of R661W viability test by CCK‐8 assay (*n* = 3).

To comprehensively evaluate the antioxidant properties of PDA@GelCA and Cur@PDA@GelCA, we compared results at the same concentration of hydrogen peroxide, but with different concentrations of PDA and Cur@PDA nanoparticles (50, 100, and 200 µg). **Figure** **5**a demonstrates that in the presence of pure GelCA hydrogel, RGC‐5 cells exhibited resorufin signals similar to the H_2_O_2_ group. However, the results for PDA@GelCA and Cur@PDA@GelCA showed that as the hydrogel contained a higher concentration of nanoparticles, the resorufin signal weakened. Specifically, the Cur@PDA@GelCA groups containing 100 and 200 µg of nanoparticles displayed significantly weaker resorufin signals compared to the corresponding PDA@GelCA groups with the same nanoparticle weight. Figure 5b quantifies the intensity of the resorufin signal in RGC‐5 cells. The results indicated that the resorufin signal intensity of the pure GelCA group did not significantly differ from the H_2_O_2_ group, whereas PDA@GelCA and Cur@PDA@GelCA groups exhibited differences. As the concentration of nanoparticles increased in both groups, the resorufin signal intensity decreased. Comparing the PDA@GelCA and Cur@PDA@GelCA groups, there was no significant difference in resorufin signal between the two groups with 50 and 200 µg nanoparticles. However, at 100 µg nanoparticle concentration, both groups exhibited a significant difference in resorufin signal intensity, indicating a distinction in antioxidant properties between PDA@GelCA and Cur@PDA@GelCA hydrogels under 200 µM hydrogen peroxide conditions.

Furthermore, to test the in vitro biocompatibility of the three hydrogels, CCK‐8 assay and live & dead fluorescence staining were performed to assess the viability of RGC‐5 and R661W cells. The results from the CCK‐8 assay, as shown in Figure 5c,d, indicate that there were no significant differences in cell viability between the control group and the groups cultured with the extract of GelCA, PDA@GelCA, or Cur@PDA@GelCA hydrogels after 1 and 2 days of cultivation. This suggests that the three hydrogels exhibited no significant cytotoxicity. Supporting these results, Figure S10a,b, Supporting Information, displays the live and dead staining results of RGC‐5 and R661W cells after 2 days of cultivation in the presence of the hydrogel extracts. The obtained results aligned with the observed outcomes from the CCK‐8 assay, demonstrating that no significant disparity in cell viability was observed between the groups cultured with the extract of the three hydrogels and the control group.

### 2.5 In Vivo Bio‐Compatibility Performance of Nanoparticle‐Loaded Hydrogel

**Figure 6:**
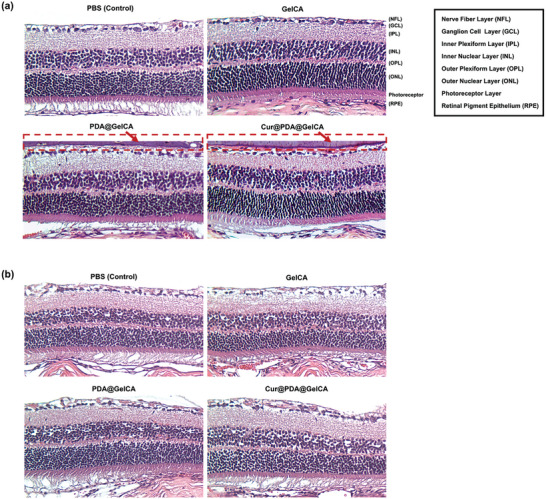
a) Detected by dihydroethidium (DHE) fluorescence staining was the production of superoxide anion (scale bars: 100 µm). b) Immunofluorescent staining of retinal cells with Brn3C, and nuclear staining with Hoechst33342 (scale bars: 100 µm). c) Quantitative analysis of the relative fluorescence intensity of DHE was conducted along with representative imaging and ROS level analysis using ANOVA in retinal ganglion cell (RGC) sections (*n* = 3). d) Quantitative analysis of the Brn3C^+^ Cells was conducted along with representative imaging (*n* = 3).

To assess the in vivo compatibility of GelCA, PDA@GelCA, and Cur@PDA@GelCA hydrogels, we conducted intravitreal injections in healthy mice, and retinal samples were collected for evaluation after 7 and 14 days of injection. From the H&E staining results depicted in **Figure** **6**a, it was observed that both PDA@GelCA and Cur@PDA@GelCA hydrogels exhibited excellent adhesion outside the nerve fiber layer (NFL) (highlighted in red boxes) within the first 7 days post‐injection, whereas the group injected with pure GelCA hydrogel did not show adhesion outside the NFL. This suggests that the incorporation of PDA and Cur@PDA nanoparticles enhanced the tissue surface adhesion of the hydrogels, owing to the strong pi–pi stacking and hydrogen bonding capabilities of PDA nanoparticles. Furthermore, the groups injected with GelCA, PDA@GelCA, and Cur@PDA@GelCA hydrogels showed no significant differences compared to the control group (PBS) in the retinal tissue. These findings indicate that the injection of hydrogels did not result in cell loss, tissue atrophy, or inflammatory reactions, both on day 7 and day 14 post‐injection (Figure 6a,b). The retinal tissue maintained the integrity of major functional cell layers such as the ganglion cell layer (GCL), inner nuclear layer (INL), and outer nuclear layer (ONL). Hence, GelCA, PDA@GelCA, and Cur@PDA@GelCA hydrogels demonstrate excellent in vivo compatibility with retinal tissue, with no evidence of tissue toxicity effects. These results highlight the potential biomedical applications of these hydrogels in ophthalmology and regenerative medicine.

### 2.6 In Vivo Antioxidative Performance of Nanoparticle‐Loaded Hydrogel with ONC Model

To investigate the antioxidant performance of PDA@GelCA and Cur@PDA@GelCA in vivo, we utilized the ONC model to simulate retinal oxidative stress in mice. The ONC model is commonly employed to assess the antioxidant properties of retinal tissue, as it induces an increase in intracellular ROS production, triggering a series of cellular oxidative stress responses. This process can activate oxidases, resulting in elevated ROS generation.^[^ ^71^, ^72^ ^]^ Following the ONC procedure, intravitreal injections of GelCA, PDA@GelCA, and Cur@PDA@GelCA were administered to the retinal tissue to evaluate the antioxidant characteristics of the samples.

**Figure 7:**
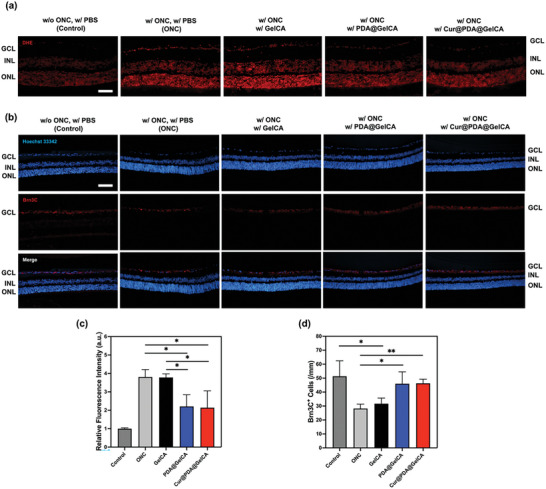
a) Detected by dihydroethidium (DHE) fluorescence staining was the production of superoxide anion (scale bars: 100 µm). b) Immunofluorescent staining of retinal cells with Brn3C, and nuclear staining with Hoechst33342 (scale bars: 100 µm). c) Quantitative analysis of the Brn3C^+^ Cells was conducted along with representative imaging (*n* = 3). d) Quantitative analysis of the relative fluorescence intensity of DHE was conducted along with representative imaging and ROS level analysis using ANOVA in retinal ganglion cell (RGC) sections (*n* = 3).

First, tissue staining was performed using dihydroethidium (DHE), which is commonly used to assess the oxidative state of retinal tissue. DHE is oxidized by ROS to form a fluorescent dye called ethidium. Subsequently, ethidium binds to DNA, leading to the generation of red fluorescence in the cell nucleus.^[^ ^73^, ^74^ ^]^ The DHE staining results, as shown in **Figure** **7**a, reveal an increase in oxidative stress in retinal tissue following ONC treatment. The ONC group exhibited a higher fluorescent intensity level compared to the control group, indicating the impact of oxidative stress on the GCL, ONL, and INL after ONC treatment. However, in the groups treated with the three hydrogels after ONC, the GelCA hydrogel group exhibited significant fluorescence intensity similar to the ONC group. On the other hand, the PDA@GelCA and Cur@PDA@GelCA hydrogel groups showed intermediate fluorescence intensity between the ONC and control groups. Furthermore, the quantitative analysis of fluorescence intensity, as shown in Figure 7c, indicates no significant difference between the GelCA group and the ONC group, suggesting that GelCA hydrogel alone had a limited impact on tissue antioxidant properties. In contrast, both the PDA@GelCA and Cur@PDA@GelCA hydrogel groups exhibited significant differences compared to the ONC group, indicating their ability to further alleviate oxidative damage in the tissue following ONC treatment. However, based on the DHE staining, it was not possible to differentiate the antioxidant protective properties between the PDA@GelCA and Cur@PDA@GelCA groups.

In addition, the Brn3C antibody was employed to label ganglion cells in retinal specimens collected after in vivo material injection. Brn3C is a specific antibody for RGCs. Figure 7b shows the results of tissue nuclear staining and immunofluorescent staining of Brn3C‐positive cells. The control group received PBS injection without optic nerve crush (ONC), and the Brn3C immunostaining showed no significant reduction or atrophy in the GCL. The ONC group received PBS injection after ONC, and a significant decrease in Brn3C‐positive cells was observed. In the group where GelCA was injected after ONC, the number of Brn3C‐positive cells was similar to the ONC group, indicating a significant reduction in ganglion cells. Finally, in the groups injected with PDA@GelCA and Cur@PDA@GelCA hydrogels after ONC, the number of Brn3C‐positive cells was comparable to the control group, demonstrating that the hydrogels loaded with PDA and Cur@PDA nanoparticles can further inhibit excessive oxidative stress in retinal tissue after ONC and protect against apoptosis in the GCL. Figure 7d presents the statistical analysis of the number of cells per millimeter in the GCL from fluorescent images. The results indicate that ONC indeed decreases the cell count in the GCL, but the injection of PDA@GelCA and Cur@PDA@GelCA hydrogels effectively mitigated the reduction in cell count.

## 3 Conclusion

In this study, we successfully grafted cinnamic acid moieties onto gelatin to create GelCA macromers with a high degree of substitution (91%). We then crosslinked the modified GelCA hydrogel using photo‐initiated dimerization under 365 nm UV irradiation, capitalizing on the alkene functional group (C═C) in cinnamic acid. This modification introduced π–π stacking abilities to the hydrogel, enabling it to adsorb drugs with similar stacking capabilities. We achieved the synthesis of PDA) nanoparticles, utilizing their π–π stacking and hydrogen bonding properties to load curcumin. Comparative assessments were conducted on both types of nanoparticles, highlighting the enhanced efficacy of Cur@PDA nanoparticles in inhibiting ROS, such as DPPH• free radicals and superoxide, compared to pure PDA nanoparticles. However, the limited bioavailability of curcumin due to its lipophilicity led us to load it onto hydrophilic PDA nanoparticles. The in vitro experiments confirmed the lack of cytotoxicity for nanocomposite hydrogels, namely PDA@GelCA and Cur@PDA@GelCA. These hydrogels protected retinal ganglion‐derived cells (RGC‐5) from oxidative stress damage induced by H_2_O_2_. In vivo injections of GelCA, PDA@GelCA, and Cur@PDA@GelCA hydrogels into retinal tissues showed no tissue atrophy or cell loss after 7 and 14 days, indicating strong in vivo biocompatibility. Furthermore, the hydrogels, particularly Cur@PDA@GelCA, effectively prevented the generation of high levels of ROS signals following an ONC model, preserving RGC viability. In a concise summary, Cur@PDA@GelCA has demonstrated outstanding biocompatibility and proven effective in safeguarding retinal tissue against oxidative stress‐induced damage. Its remarkable adhesive properties and long‐lasting effects offer exciting potential for acute neuroprotection in the context of inner retinal injuries. Furthermore, when integrated with stem cell therapy, this combination holds promise for fostering neuroregeneration in the foreseeable future.

## 4 Experimental Section

### Materials

Gelatin type B (Sigma‐Aldrich, USA, gel strength: ≈225 g Bloom), trans‐cinnamic acid (Alfa Aesar, USA, purity: 99+%), EDC (ACROS Organics, USA, purity: 95+%), lithium phenyl‐2,4,6‐trimethylbenzoylphosphinate (LAP, Sigma‐Aldrich, Germany, purity: 95+%), dopamine hydrochloride (Alfa Aesar, USA, purity: 99%), ammonium hydroxide solution (Sigma‐Aldrich, Germany, 28% NH_3_ in H_2_O, 99.99+% trace metals basis), curcumin (Sigma‐Aldrich, Germany, purity: 98%), glycine (ACROS Organics, USA, purity: 99%), sodium bicarbonate (Sigma‐Aldrich, Germany), 2,4,6‐trinitrobenzene sulfonic acid (CSL, AU), QuantiProTM BCA assay kit (Sigma‐Aldrich, USA), bovine serum albumin (BSA, Sigma‐Aldrich, USA), 2,2‐diphenyl‐1‐picrylhydrazyl (Alfa Aesar, USA, purity: 95% ), Riboflavin (Sigma‐Aldrich, Germany, purity: 98+%), NBT (Sigma‐Aldrich, Germany, purity: 90+%), l‐methionine (Sigma‐Aldrich, Germany, purity: 98+%), Dulbecco's modified Eagle's medium/high glucose (DMEM/HG, HyClone, USA), DAPI staining solution (Abcam, USA), Alexa FluorTM Phalloidin (Invitrogen, USA), and 2′,7′‐dichlorofluorescin diacetate (Sigma‐Aldrich, USA).

### Fabrication of GelCA Macromer

The modification of GelCA followed a high degree of substitution method based on a previous protocol.^[^ ^37^ ^]^ The molar ratio of gelatin to cinnamic acid to EDC was set as 1:9:90. The construction of GelCA was achieved by utilizing gelatin (type B), cinnamic acid, and the crosslinker, EDC. Initially, 0.0035 mol of cinnamic acid was dissolved in 20 mL of 2 n NaOH solution, with the pH of the solution being precisely adjusted to 8. Subsequently, 0.043 mol of EDC was incorporated into 60 mL of PBS within a 4 °C environment, ensuring the uniform dispersion of EDC within the PBS solution. Following this, the two solutions were combined and subjected to a 30‐min stirring process at 4 °C. In the third phase, 5 g of gelatin (type B) was introduced into 50 mL of PBS and heated to 60 °C to ascertain the even dispersion of gelatin within the solution. Post‐cooling to ambient temperature, the previously prepared 4 °C mixed solution was added to the gelatin solution, and this combination was stirred for a period of 24 h at room temperature. Upon completion, 6–8 kDa dialysis bags were utilized to separate the reactant chemicals from the GelCA macromer. As a final step, the GelCA macromer was lyophilized and securely stored within a drying cabinet.

### Fabrication of Curcumin‐Loaded Polydopamine (Cur@PDA) Nanoparticles

For the production of PDA nanoparticles, 1 g of dopamine hydrochloride was first dissolved in 40 mL of DI water. The solution was then combined with a mixture of 4 mL of ammonium hydroxide, 160 mL of water, and 80 mL of ethanol. The mixture was stirred for 24 h at room temperature to polymerize dopamine. The solution was then centrifuged and lyophilized to separate and collect the PDA nanoparticles.

To synthesize curcumin‐loaded PDA nanoparticles (Cur@PDA), 10 mg of curcumin was thoroughly dispersed in a 2 mL methanol solution. Subsequently, 10 mg of PDA nanoparticles were prepared in 8 mL of DI water. The curcumin solution was then gradually introduced into the PDA nanoparticle mixture. Following this, an additional 6 mL of methanol was added. The mixture was allowed to react for 24 h. The resulting Cur@PDA nanoparticles were collected via centrifugation, cleaned with appropriate washing steps, and finally, lyophilized for future use.

### Fabrication of Cur@PDA‐Loaded GelCA Hydrogel

First, a specified amount of Cur@PDA nanoparticles were dissolved in 900 µL of DI water, and the nanoparticles were evenly dispersed in the solution using an ultrasonic homogenizer (Sonic Ruptor 4000). Next, 50 mg of GelCA was dissolved in the solution containing the nanoparticles and mixed with 100 µL of 0.6 wt% LAP solution. Finally, the mixture was exposed to 25 mW cm^−2^ of UV light (365 nm) for 3 min, causing the GelCA to crosslink under the photoinitiator (LAP) and UV irradiation.

### Fourier‐Transform Infrared Spectrometry Analysis

The chemical structure and bonding of samples were determined using FTIR spectroscopy (Perkin Elmer spectrum 100, USA). First, the sample was dried and ground with potassium bromide (KBr) in a 1:100 weight ratio, then the mixture was pressed into a thin film shape using the pellet press. The spectrum of the sample was recorded with a resolution of 4 cm^−1^ and scanned 16 times in the wavenumber range between 4000 and 450 cm^−1^.

### Nuclear Magnetic Resonance Spectrometry Analysis

1 mg of cinnamic acid, gelatin, and modified gelatin (GelCA) were dissolved in 600 µL D2O with 1 w/v% NaOH, and their ^1^H NMR spectra were recorded using a 500 MHz FT‐NMR instrument (Bruker AVIII‐500 from Germany) with a process controller.

### Measurement of the Mechanical Properties of Hydrogels

In order to evaluate the mechanical properties of the hydrogel, both rheological and compression tests were conducted. For the rheological examination, a rheometer (HR‐2 system, TA Instrument) was utilized to measure the viscoelastic properties of the hydrogel through the photo‐crosslinking process, the shear‐thinning properties of the hydrogel to demonstrate the injectibility, and test the self‐healing property of hydrogel. For the test of the viscoelastic properties, the precursor solution of the hydrogel was subjected to UV irradiation at 25 mW cm^−2^ (365 nm) at room temperature using an 8 mm‐diameter plate geometry set at an 0° angle, and an oscillation with a fixed strain of 1% and an angular frequency of 10.0 rad s^−1^ was applied. The storage modulus and loss modulus were recorded throughout the photo‐crosslinking process and the measurement was halted when the storage modulus reached stability. The shear‐thinning property was evaluated by the flow sweep with 300 µL complete gelation hydrogels under the shear rate from 1 to 100 s^−1^ at 37 °C using a 20 mm‐diameter plate geometry set at a 0° angle. The self‐healing test was also conducted by continuous step strain sweep with 300 µL complete gelation under low strain (0.1% strain) and high strain (1000% strain) at 1 rad s^−1^ at 37 °C using a 20 mm‐diameter plate geometry set at a 0° angle.

In addition, a dynamic compression test was performed to further probe into the mechanical characteristics of the hydrogels. The precursor solution was injected into a mold of specific dimensions and exposed to UV irradiation at 25 mW cm^−2^ (365 nm) to achieve complete crosslinking into hydrogels. After the full saturation of photo‐crosslinking was achieved for a variety of hydrogels, they were subjected to compression using an 11.94 mm compressor of texture analyzer at a rate of 6 mm min^−1^. Data were recorded at a rate of 10 per second, the calculation of the stress experienced by the compressor and the strain rate of hydrogels.

### Measurement of Cinnamic Acid Substitution on Gelatin

The TNBS assay was used to determine the degree of amine group substitution on GelCA. The lyophilized GelCA was dissolved in DI (0.1 w/v%) water and fivefold diluted with a sodium bicarbonate buffer (0.1 m, pH 9.8). 40 µL 2,4,6‐trinitrobenzene sulfonic acid was also diluted with 19.96 mL DI water and then reacted with GelCA for 1 h. The reaction was then stopped with 250 µL HCl (0.1 n) for 30 min. The absorbance was measured at 353 nm. In this assay, 2.5 mm glycine solution was prepared and serial diluted to serve as the standard, and gelatin was used as the control.

The degree of amine group substitution on GelCA (%) was calculated using the following formula

(1) $$\mathrm{Degree}\;\;\mathrm{of}\;\;\mathrm{substitution}\;\;\left(\%\right)=\left(1-\frac{C_{\mathrm{amine},\;\mathrm{GelCA}}/C_{\mathrm{GelCA}}}{C_{\mathrm{amine},\;\mathrm{Gelatin}}/C_{\mathrm{Gelatin}}}\right)\times 100\%$$

where *C*_amine, GelCA_ is the concentration of the amine chemical group in the GelCA solution, *C*_amine, Gelatin_ is the concentration of the amine chemical group in Gelatin solution, *C*_GelCA_ is the concentration of GelCA solution, and *C*_Gelatin_ is the concentration of the Gelatin solution.

### Swelling Test of Hydrogels

The swelling ratio of hydrogels was assessed through incubation in DI water. First, 400 µL of fully photo‐crosslinked hydrogels were prepared, followed by lyophilization to ensure the complete removal of water content from the hydrogels. Subsequently, the lyophilized hydrogels were immersed in 5 mL of DI water, and their weights were measured at specific time points.

(2) $$\mathrm{Swelling}\;\;\mathrm{ratio}\left(\%\right)=\frac{W_{w}/W_{D}}{W_{D}}\times 100\%$$

where *W*_w_ is the weight of the wet hydrogel and  *W*_D_ is the weight of the dry hydrogel.

### Long‐Term Dissolution and Short‐Term Degradation Test of Hydrogels

The long‐term degradation of hydrogels was investigated by incubating 400 µL of fully‐swollen hydrogels in 3 mL of pure DMEM/HG medium at 37 °C. At specific time points, the medium was removed, and the weight of the hydrogels was recorded. For the short‐term degradation test, collagenase type 1 was utilized as the enzyme. Specifically, the 400 µL fully‐swollen hydrogels were immersed in a 3 mL enzyme solution (10 U mL^−1^) and incubated at 37 °C. The degradation solution was then removed from the samples, and the weight of the hydrogel was recorded at a specific time point.

(3) $$\mathrm{Remaining}\;\;\mathrm{percentage}\left(\%\right)=\frac{W_{D}-W_{S}}{W_{D}}\times 100\%$$

where *W*_S_ is the weight of the hydrogel and dish.  *W*_D_ is the weight of dry hydrogel and dish.

### Adhesion Test of Hydrogels on Porcine Skin

To quantitatively assess the tissue adhesion strength of hydrogels, two lap‐shear adhesion tests were conducted, following ASTM F2255‐05 guidelines with some adaptations. In brief, 15 mm × 15 mm squares were obtained from porcine skin tissue and affixed to two glass slides using cyanoacrylate glue. Subsequently, 20 uL of hydrogel was applied in situ between two skin tissues, polymerizing it with 365 nm UV irradiation for 5 min. A 2500 g weight was placed on each assembly and allowed to rest for 10 min. The lap‐shear strengths of the hm‐PVA films were determined using a texture analyzer at a tracking speed of 0.1 mm s^−1^. All the procedural details are illustrated in Figure S7, Supporting Information.

### Observation Using a Scanning Electron Microscope

An exploration of the sample's surface morphology was conducted utilizing SEM. In preparation for SEM, samples were subjected to a thorough drying process, ensuring the elimination of any residual surface solution. Thereafter, the samples were coated with platinum metal particles employing a metal sputtering apparatus. SEM examination was executed with the aid of a NovaTM NanoSEM 230 (Thermo, Waltham, MA, USA). The meticulous observation of the sample surfaces was performed at 5 kV within a high vacuum chamber. Electron signals were proficiently detected via an Everhart–Thornley detector (ETD) and a through‐the‐lens detector (TLD). Subsequent to obtaining the SEM images, they were processed and comprehensively analyzed through ImageJ software, a robust tool in the realm of image processing.

### Observation of Porosity and Pore Morphology in Hydrogels

Micro‐CT was employed to investigate the internal porous structure of the hydrogels. 500 µL of hydrogels were fully lyophilized and subjected to Micro‐CT imaging using X‐rays. This technique enabled a detailed slice‐by‐slice analysis of the microstructure of the hydrogels.

The mercury porosimeter (AutoPore IV 9520, USA) was utilized to assess the porosity and pore size distribution of the hydrogels. The hydrogels, fully lyophilized, were infiltrated with mercury at an environmental temperature of 25 °C and relative humidity of 50%. The porosity of the hydrogels was determined by analyzing the intrusion curve obtained from the mercury porosimeter.

### Dynamic Light Scattering Analyzer Analysis

A comprehensive characterization of the nanoparticles, encompassing both size and surface charge, was performed utilizing a Zetasizer (Zetasizer‐ZS90 Plus, Malvern, UK). To mitigate any potential interference from multiple scattering during data interpretation, the nanoparticles were uniformly dispersed either in DI water or a 2% ethanol solution. This was followed by diluting the solution to an appropriate concentration, carried out at room temperature, to ensure consistency in measurements. Particle size and zeta potential were measured employing two sophisticated techniques, photon correlation spectroscopy and electrophoretic light scattering, respectively. To ensure reliability and precision in data acquisition, all measurements were recorded in triplicate.

### Quantitative Test for Polydopamine Nanoparticles Loaded with Curcumin

Upon completing the curcumin‐loading process onto PDA nanoparticles, the nanoparticles were centrifuged at a speed of 10 000 revolutions per minute for a duration of 15 min to ensure comprehensive sedimentation. The resulting supernatant was carefully collected and subjected to a 100‐fold dilution. The optical density of the supernatant at 425 nm was subsequently measured utilizing a UV/vis spectrophotometer (Cary 300nc, Agilent, USA). To facilitate precise quantitative analysis, a standard curve was meticulously established. This was achieved by preparing a methanol solution containing curcumin in a series of calibrated dilutions. Through these robust procedures, the accurate determination of curcumin loading on the PDA nanoparticles was ensured.

The curcumin loading rate on PDA nanoparticles was calculated using the following formula

(4) $$\begin{array}{ccc} \mathrm{Curcumin}\;\;\mathrm{loaded}\;\;\mathrm{rate}\left(\%\right) & = & \frac{M_{\mathrm{curcumin},\;\mathrm{initial}}-C_{\mathrm{curcumin},\;\mathrm{final}}\times V_{\mathrm{final}}}{M_{\mathrm{PDA},\;\mathrm{initial}}}\\ & & \times \;100\% \end{array}$$

where *M*_curcumin, initial_ is the mass of curcumin in the initial solution, *C*_curcumin, final_ is the concentration of unloaded curcumin in the final supernatant, *V*_final_ is the volume of the final supernatant, and *M*_PDA, initial_ is the mass of PDA nanoparticles in the initial solution.

### Observation Using a Transmission Electron Microscope

The detailed morphology of PDA nanoparticles was investigated using a TEM. Furthermore, the morphological alterations on the nanoparticle surfaces following curcumin adsorption (Cur@PDA) were comparably evaluated. In preparation for TEM analysis, nanoparticle samples were uniformly dispersed in DI water. A precise volume of 7 µL of the sample solution was then adsorbed onto a copper mesh and allowed to rest for a period of 5 min. Any residual solution was meticulously removed using filter paper to maintain the integrity of the sample. Subsequently, the nanoparticles were stained using a 2 w/v% uranyl acetate (UA) solution, enhancing the visibility and contrast under TEM. TEM observations were carried out using a high‐resolution H‐7650 microscope (Hitachi, Japan).

### Testing the ROS Scavenging Efficiency of Materials

The efficacy of the nanoparticles in scavenging free radicals was determined by incubating 1 mL of a freshly prepared 1,1‐diphenyl‐2‐picrylhydrazyl (DPPH•) solution (with a concentration of 100 µg DPPH•/mL in methanol) with 2 mL of a nanoparticle suspension at various concentrations, namely 0, 10, 20, 40, and 80 µg mL^−1^ in methanol. This incubation was conducted for 30 min at a controlled temperature of 31 °C, in an environment devoid of light. Concurrently, the scavenging efficiency of hydrogels against DPPH• radicals was evaluated by incubating 200 µL of hydrogels in 1 mL of DPPH• solution (100 µg mL^−1^ in methanol) for 1 h. For the hydrogels PDA@GelCA and Cur@PDA@GelCA, NP concentrations were set at 40 and 80 µg per 100 µL of (5 wt%) GelCA hydrogel, respectively. These conditions were also maintained at a constant temperature of 31 °C, with precautions taken to avoid light exposure.

After the incubation with the DPPH• solution, absorbance at 517 nm was meticulously measured using a UV–vis spectrophotometer (Cary 300nc, Agilent, USA). This measurement facilitated the subsequent quantification of the residual DPPH• radicals. Utilizing this methodology, the free‐radical‐scavenging potential of the nanoparticles being studied could be effectively assessed.

The DPPH• inhibition rate of nanoparticles was calculated using the following formula

(5) $$\mathrm{Inhibition}\left(\%\right)=\left(1-\frac{C_{\mathrm{DPPH}•,\;\;x}}{C_{\mathrm{DPPH}•,\;\;0}}\right)\times 100\%$$

where *C*_DPPH•,  *x*_ is the concentration of DPPH• in solution with nanoparticles and hydrogels, and *C*_DPPH•,  0_ is the concentration of DPPH• in solution in the absence of both nanoparticles and hydrogels.

The superoxide scavenging efficacy of the materials was evaluated by employing the NBT assay. For this assay, each solution contained riboflavin (20 µm), methionine (12.5 mm), and NBT (75 µm) in PBS (25 mm, pH 7.4). These solutions were then supplemented with varying concentrations of PDA and Cur@PDA NPs ranging from 0 to 80 µg mL^−1^, alongside different NP concentrations of GelCA, PDA@GelCA, and Cur@PDA@GelCA hydrogels to ascertain their capacity to scavenge superoxide radicals.

These mixtures were exposed to 365 nm UV radiation for 5 min. Subsequently, the absorbance of the mixtures was measured. A sample containing riboflavin, methionine, and NBT served as the negative control, while a sample containing riboflavin, methionine, and NBT after exposure to illumination was designated as the positive control. All experiments were conducted in darkness without illumination. The inhibition percentage was calculated using the following formula.

The superoxide inhibition rate of nanoparticles was calculated using the following formula

(6) $$Inhibition\left(\%\right)=\left(\frac{A_{0}-A_{n}}{A_{p}-A_{n}}\right)\times 100\%$$

*A*_0_, *A*_n_, and *A*_p_ are the absorbance of the treated samples, negative control, and positive control, respectively.

### In Vitro Quantitative Test for Release of Curcumin from Cur@PDA@GelCA Hydrogel

2 mg of Cur@PDA NPs, containing ≈0.230 mg of curcumin, were incorporated into 2 mL of 5% GelCA hydrogel. This mixture was then transferred to a sample vial containing 5 mL of PBS. The vial was subsequently placed in an incubator set at 37 °C to initiate in vitro drug release investigations. At predefined time intervals (0.25, 1, 2, 4, 8, 24… 168 h), a 250 µL aliquot of the media was withdrawn and replaced with an equivalent volume of fresh PBS. The samples were filtered through a 0.45‐µm filter and subjected to analysis using a spectrophotometer, measuring the released curcumin at 425 nm.

### Sterilization Process of Hydrogels

To ensure the sterility of GelCA, PDA@GelCA, and Cur@PDA@GelCA hydrogels used in both in vitro and in vivo experiments, several steps are employed. Initially, GelCA hydrogel was prepared as a solution of the desired concentration. Subsequently, it was heated to 40 °C and passed through a 0.22 µm sterile filter for sterilization. In the case of the PDA@GelCA and Cur@PDA@GelCA groups, PDA and Cur@PDA nanoparticles were sterilized under UV irradiation at 25 mW cm^−^^2^ for 30 min. Following this, they were introduced into the previously sterilized GelCA solution. Finally, a 0.06% LAP solution was added, and gelation was performed under sterile conditions in a sterile laminar flow hood to ensure the sterility of the materials.

### In Vitro Cell Compatibility Test of Hydrogels

CCK‐8 assay and Live/Dead assay were employed to evaluate the in vitro biocompatibility of the hydrogels. According to the experimental protocol specified in ISP10993‐5, the CCK‐8 assay was used for quantitative analysis of cell viability, while the Live/Dead assay was used for qualitative analysis.

To prepare for the CCK‐8 assay, 400 µL of hydrogels were immersed in DMEM/HG medium. At specific time points (4, 24, and 48 h), 150 µL of extract was collected. RGC‐5 and R661W cells were separately cultured with 150 µL of the extract, while the control group was treated with growth medium as a counterpart. CCK‐8 buffer mixed with 100 µL of growth medium was added to each well at 4, 24, and 48 h. The plates were then incubated at 37 °C, 5% CO_2_ for 2 h. If cells were highly active, the medium would change color from pink to orange. Subsequently, 100 µL of the reacted medium was transferred to a new 96‐well plate, and the absorbance was measured at 450 nm. The absorbance values of three replicates were normalized and compared to the average value of the control groups.

Additionally, the Live/Dead stain was performed on the groups incubated with the extract of hydrogels for 48 h to conduct qualitative analysis. The Live/Dead assay involved staining the cells with Calcein AM (live dye) and Ethidium homodimer I (dead dye) diluted at a 1:500 ratio in PBS for 10 min at 37 °C. The cells were then observed directly under fluorescence microscopy.

### In Vitro Fluorescence Staining for Cellular ROS Damage

In order to assess the capacity of nanoparticle‐loaded hydrogels to mitigate ROS in vitro, RGC‐5 was utilized, a cell line derived from the mouse retina. RGC‐5 cells were extensively employed in retinal research due to their relevance in studying RGCs and associated diseases. The oxidative stress of RGC‐5 cells under the influence of hydrogen peroxide was evaluated using an AmplexTM red fluorescent reagent. The experimental procedure commenced with the preparation of pure hydrogel (GelCA) and nanoparticle‐loaded hydrogels (PDA@GelCA and Cur@PDA@GelAC), subjected to 25 mW cm^−2^ UV irradiation at 365 nm for 5 min. This step ensured the complete gelation of GelCA and adsorption of quantities of nanoparticles (50, 100, and 200 µg). Next, RGC‐5 cells were cultured on well plates, allowing ample time for cellular attachment and spreading. The culture medium was then replaced with one containing 2 µm hydrogen peroxide (H_2_O_2_) for 30 min, simulating a ROS‐rich environment for the H_2_O_2_ group. For the control, the fresh culture medium was employed. In another set of trials, the three types of hydrogels (GelCA, PDA@GelCA, Cur@PDA@GelCA) were combined with a culture medium containing 2 µm H_2_O_2_, which was subsequently exposed to the RGC‐5 cells for a duration of 30 min. Upon completion of this interval, the H_2_O_2_‐enriched culture medium was removed, and the cells were washed multiple times with PBS. Finally, the cells were stained with AmplexTM red fluorescent reagent in a reaction buffer, and visualized under an inverted fluorescence microscope (IX71, OLYMPUS, Japan) at ≈585 nm. This comprehensive investigation has offered significant insights into the ROS scavenging potential of nanoparticle‐loaded hydrogels in a cellular context.

### Establishment of Optic Nerve Crush Model and Intravitreal Injections

The ONC model was conducted in accordance with a previously reported protocol.^[^ ^75^, ^76^ ^]^ The animal protocol was approved by the National Taiwan University College of Medicine and College of Public Health, Institutional Animal Care and Use Committee (IACUC). Protocol No. #20 220 365. ICR mice were subjected to anesthesia by intraperitoneal injection of xylazine (40 mg kg^−1^). Adequate anesthesia was ensured by confirming the mice entering the abdominal respiratory stage. Under the binocular operating scope, a small incision was carefully made in the conjunctiva, beginning inferiorly to the eye and extending temporally, using spring scissors. Next, micro‐forcep was used to grasp the edge of the conjunctiva adjacent to the eye and gently retract it while rotating the globe nasally. This maneuver exposed the posterior aspect of the globe, providing a clear view of the optic nerve. To perform the crush, the exposed optic nerve was carefully grasped ≈1–3 mm from the eye using Dumont #N7 cross‐action forceps. During this step, only the pressure resulting from the self‐clamping action was applied to the nerve. After the 10‐s clamping, the optic nerve was released by removing the forceps, allowing the eye to return to its normal position. Directly following the nerve crush procedure, GelCA, PDA@GelCA, and Cur@PDA@GelCA hydrogels, pre‐crosslinked through photopolymerization and pre‐swelling to a saturated state, were intravitreally injected (≈10 µL) into the anterior segment of the retina using a 30G surgical needle (Terumo, Japan).

### In Vivo Antioxidative Performance Test of Hydrogels

In accordance with previous studies,^[^ ^44^ ^]^ the detection of ROS levels in retinas was conducted using DHE. After immediate isolation, the eyes were embedded in the OCT compound (Tissue‐Tek) and rapidly frozen using liquid nitrogen. Subsequently, unfixed cryosections of 10 µm thickness were treated with DHE solution (10 µmol L^−1^, Beyotime) and incubated for 30 min at 37 °C. Fluorescent microscope imaging was performed to capture the resulting images.

To assess the survival of RGCs, the retina sections were stained with Brn3C antibody (Proteintech, 21509‐1‐AP, 1:200). The quantification of Brn3C‐positive cells in the retinal ganglion layer was performed, and the average cell count per retina was utilized for further analysis. The density of Brn3C‐positive cells (number of cells per mm) was subsequently assessed for statistical comparisons.

### Statistical Analysis

Data were represented as means ± standard deviation (SD). Statistical analysis was performed utilizing one‐way analysis of variance (ANOVA) or Student's *t*‐test, employing the GraphPad Prism 8 software. Levels of statistical significance were predetermined as follows: non‐significant (ns) for *p* > 0.05, * for *p* < 0.05, ** for *p* < 0.01, and *** for *p* < 0.001.

## Conflict of Interest

The authors declare no conflict of interest.

## Supporting information

Supporting Information

## Data Availability

The data that support the findings of this study are available from the corresponding author upon reasonable request.
