# Supplementary material for: Injectable, Antioxidative, and Tissue‐Adhesive Nanocomposite Hydrogel as a Potential Treatment for Inner Retina Injuries
Source: Adv Sci (Weinh). 2024 Jan 17;11(11):2308635. doi: 10.1002/advs.202308635 (PMC10953571; doi:10.1002/advs.202308635)
Supplement: Supplementary file 1 — Supporting Information [file ADVS-11-2308635-s001.pdf]

ADVANCED SCIENCE Open Access

Supporting Information

for *Adv. Sci.*, DOI 10.1002/advs.202308635

Injectable, Antioxidative, and Tissue-Adhesive Nanocomposite Hydrogel as a Potential Treatment
for Inner Retina Injuries

Yi-Chen Liu, Yi-Ke Lin, Yu-Ting Lin, Che-Wei Lin, Guan-Yu Lan, Yu-Chia Su, Fung-Rong Hu, Kai-Hsiang Chang, Vincent Chen, Yi-Cheun Yeh, Ta-Ching Chen and Jiashing Yu**

Supporting Information

Injectable, Antioxidative, and Tissue-Adhesive Nanocomposite Hydrogel as a Potential Treatment for Inner Retina Injuries

Yi-Chen Liu[a], Yi-Ke Lin[b], Yu-Ting Lin[a], Che-Wei Lin[a], Guan-Yu Lan[a], Yu-Chia Su[d], Fung-Rong Hu[b,c], Kai-Hsiang Chang[a], Vincent Chen[a], Yi-Cheun Yeh[d], Ta-Ching Chen[c,e]*, Jiashing Yu[a]*

[a] Department of Chemical Engineering, National Taiwan University, Taipei 10617, Taiwan

[b] Department of Ophthalmology, College of Medicine, National Taiwan University, Taipei 100233, Taiwan

[c] Department of Ophthalmology, National Taiwan University Hospital, Taipei 100225, Taiwan

[d] Institute of Polymer Science and Engineering, National Taiwan University, Taipei 10617, Taiwan

[e] Center of Frontier Medicine, National Taiwan University Hospital, Taipei 100225, Taiwan

*Corresponding author. E-mail: jiayu@ntu.edu.tw, tachingchen@gmail.com

Keywords: retinal tissue, optic nerve, gelatin, photo-crosslinkable hydrogel, curcumin, dopamine, nanoparticles, antioxidant hydrogel, injectable hydrogel

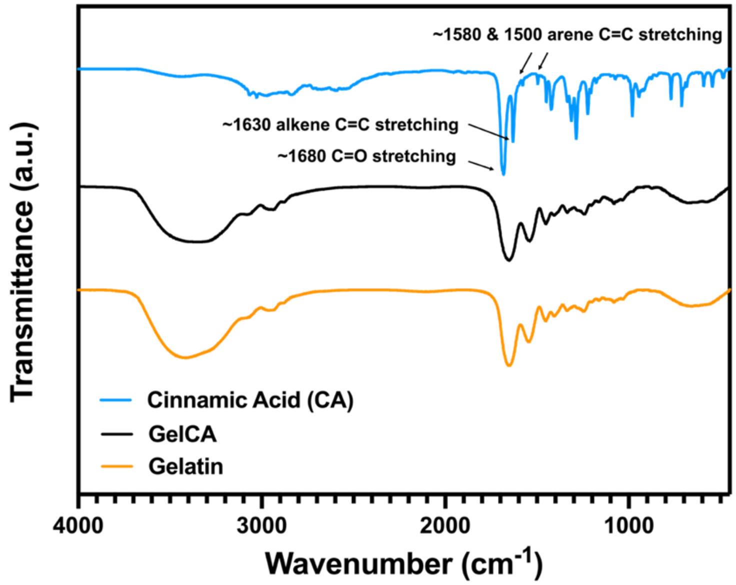

Figure S1. FTIR spectra of cinnamic acid, GelCA, and Gelatin.

(a)

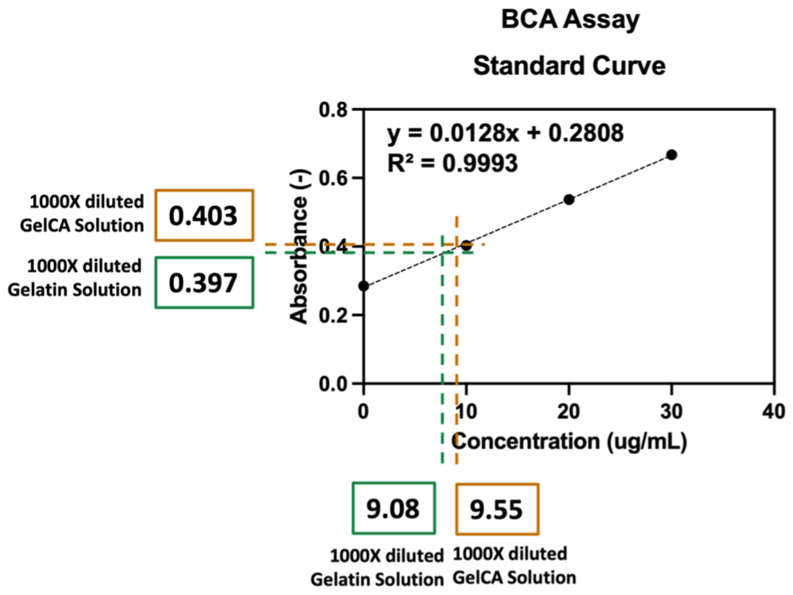

(b)

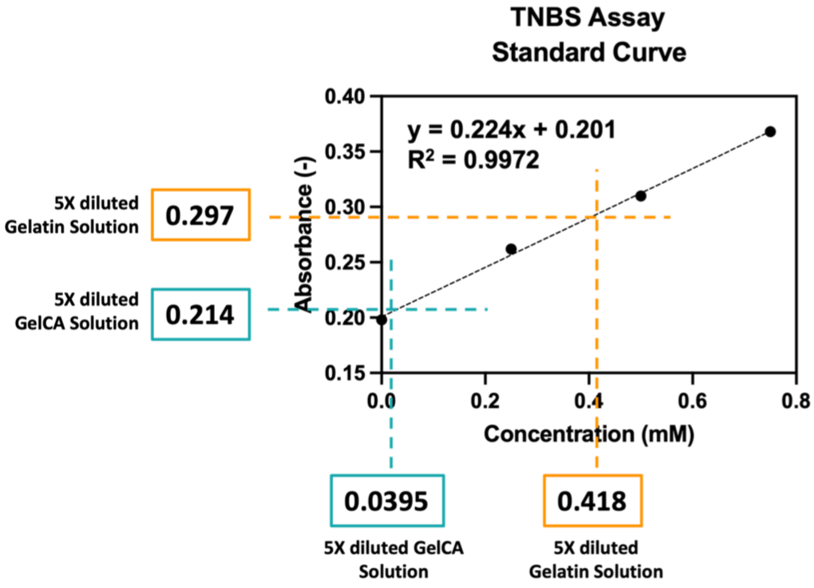

(C)

$$\textbf{Degree of substitution (\%)} = \left(1 - \frac{\frac{c_{amine,GelCA}}{c_{GelCA}}}{\frac{c_{amine,Gelatin}}{c_{Gelatin}}}\right) \times 100\%$$

$$= \left(1 - \frac{(0.0395 \times 5) \, / \, (9.55 \times 1000)}{(0.418 \times 5) \, / \, (9.08 \times 1000)}\right) \times 100\ \% = 91.0\ \%$$

Figure S2. (a) Standard curve of protein concentration in the solution for TNBS grafting test. (b) Standard curve of amine group concentration in the solution for TNBS grafting test. (c) The calculation details for determining the degree of substitution (DS) using the TNBS assay.

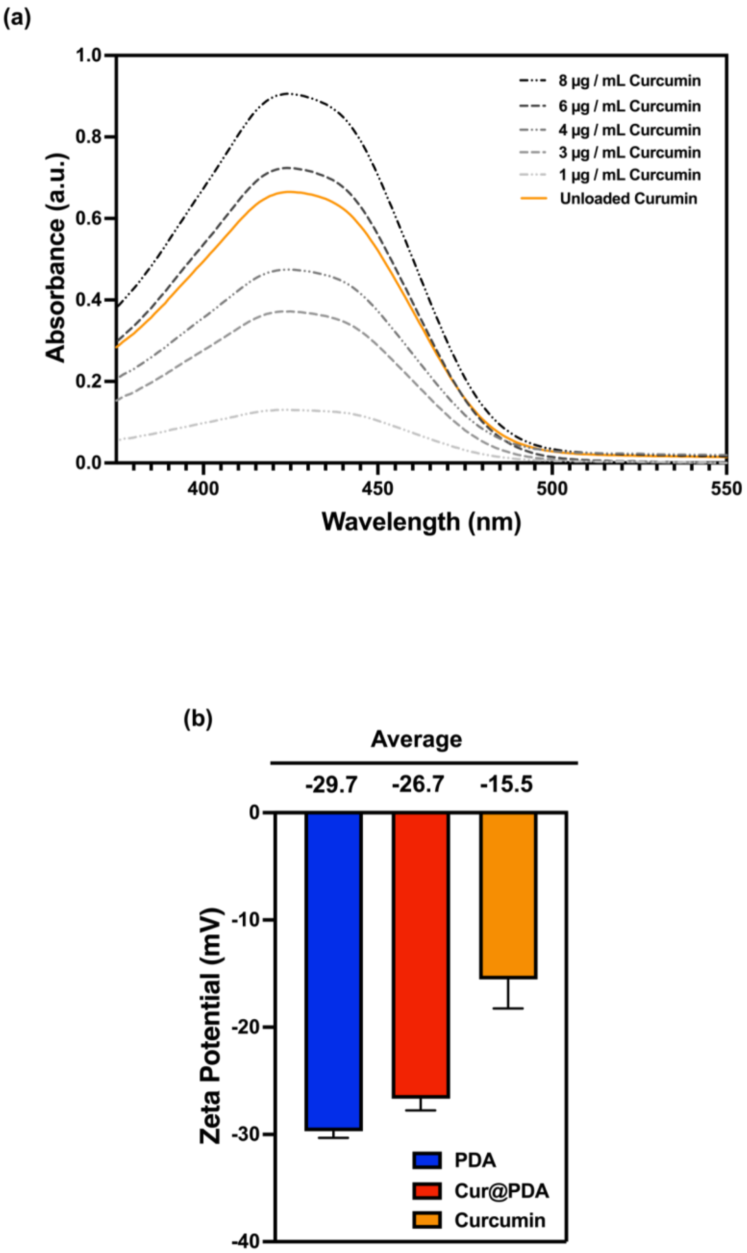

Figure S3. (a) UV-Visible spectra of curcumin solution. (b) Zeta potential of PDA, Cur@PDA nanoparticles, and Curcumin (n = 3).

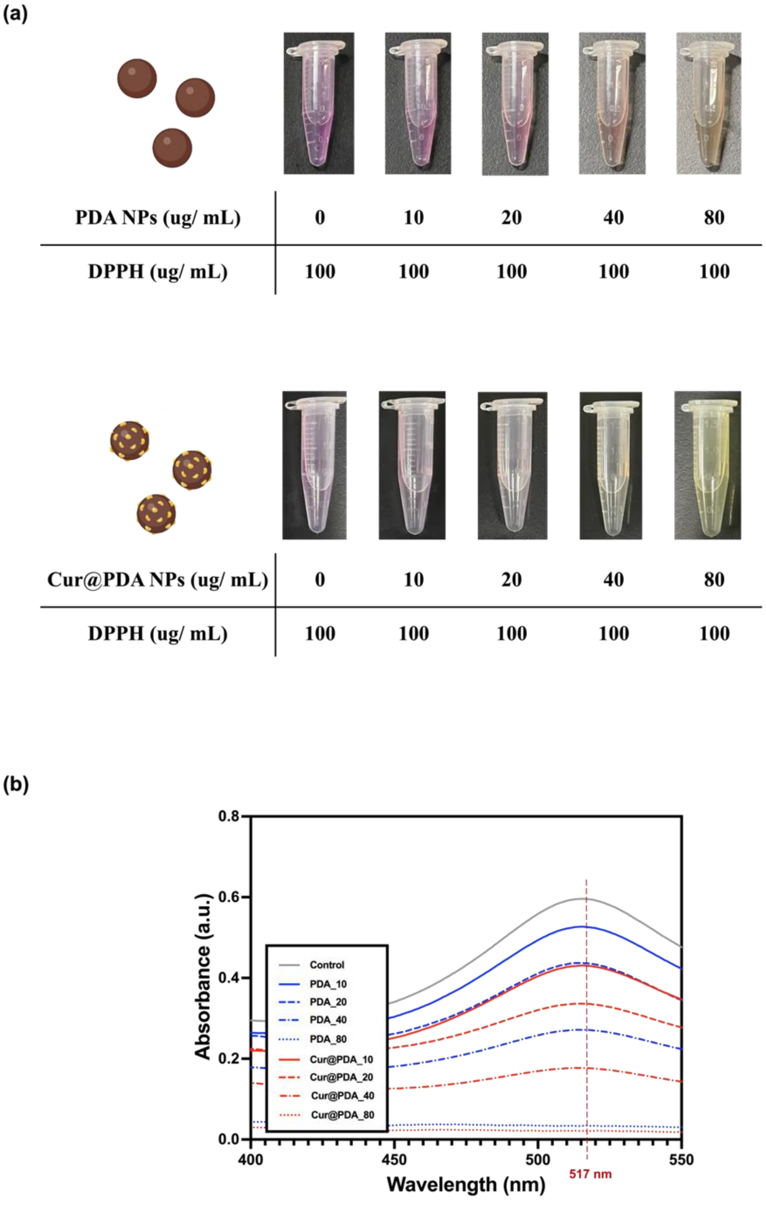

Figure S4. (a) Schematic representation of the reaction results between different concentrations of PDA NPs and Cur@PDA NPs with the DPPH• free radical solution. (b) UV-Visible spectra of DPPH free radical solution.

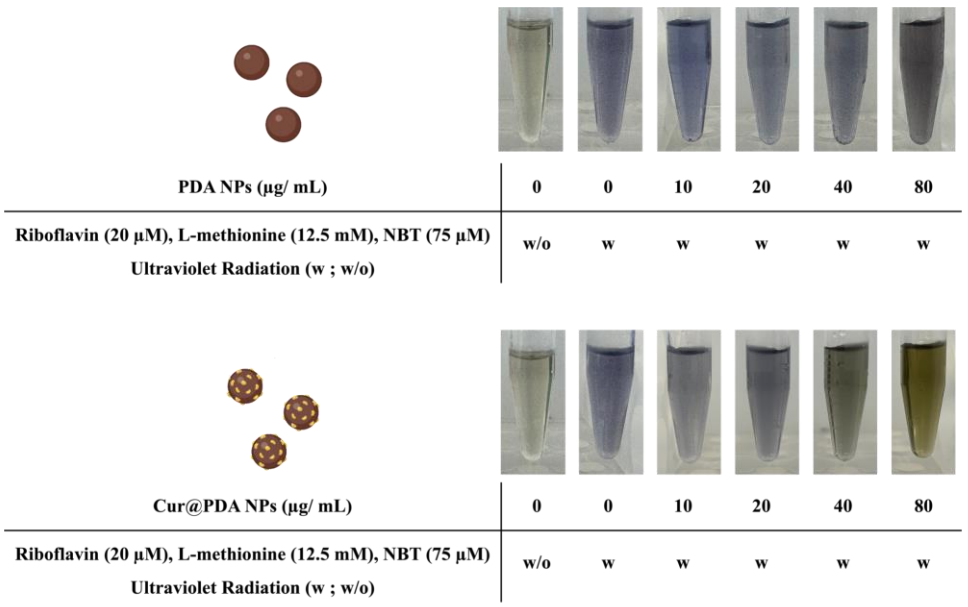

Figure S5. (a) Schematic representation of the reaction results between different concentrations of PDA NPs and Cur@PDA NPs with the $O_2^{\bullet -}$ scavenging test solution.

(a)

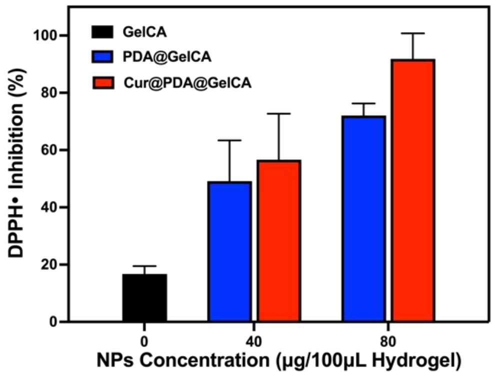

(b)

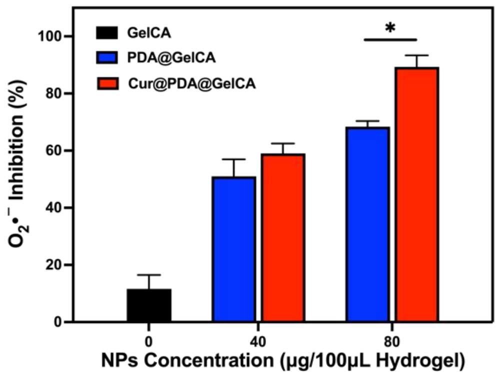

(c)

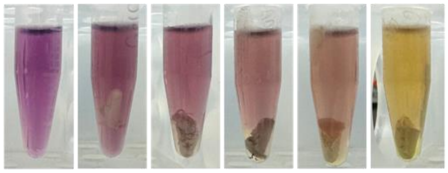

**1. 200 µL GelCA**<br>**2. 200 µL PDA@GelCA (NPs :40 µg/ 100 µL Hydrogel)**<br>**3. 200 µL PDA@GelCA (NPs :80 µg/ 100 µL Hydrogel)**<br>**4. 200 µL Cur@PDA@GelCA (NPs :40 µg/ 100 µL Hydrogel)**<br>**5. 200 µL Cur@PDA@GelCA (NPs :80 µg/ 100 µL Hydrogel)**	**N**	**1**	**2**	**3**	**4**	**5**
**DPPH (µg/ mL)**	**100**	**100**	**100**	**100**	**100**	**100**

(d)

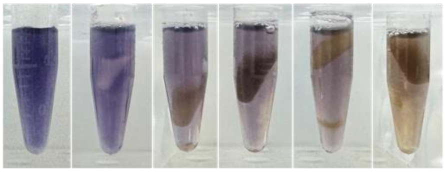

**1. 200 µL GelCA**<br>**2. 200 µL PDA@GelCA (NPs :40 µg/ 100 µL Hydrogel)**<br>**3. 200 µL PDA@GelCA (NPs :80 µg/ 100 µL Hydrogel)**<br>**4. 200 µL Cur@PDA@GelCA (NPs :40 µg/ 100 µL Hydrogel)**<br>**5. 200 µL Cur@PDA@GelCA (NPs :80 µg/ 100 µL Hydrogel)**	**N**	**1**	**2**	**3**	**4**	**5**
**Riboflavin (20 µM), L-methionine (12.5 mM), NBT (75 µM)**<br>**Ultraviolet Radiation (w ; w/o)**	**w**	**w**	**w**	**w**	**w**	**w**

Figure S6. (a) The DPPH• free radical and (b) the $O_2^{\bullet -}$ scavenging activity of hydrogels at different NP concentrations (n = 3). (c) Schematic representation of the reaction results between different hydrogels at different concentrations of NPs through the DPPH• free radical test solution and (d) the $O_2^{\bullet -}$ scavenging test solution.

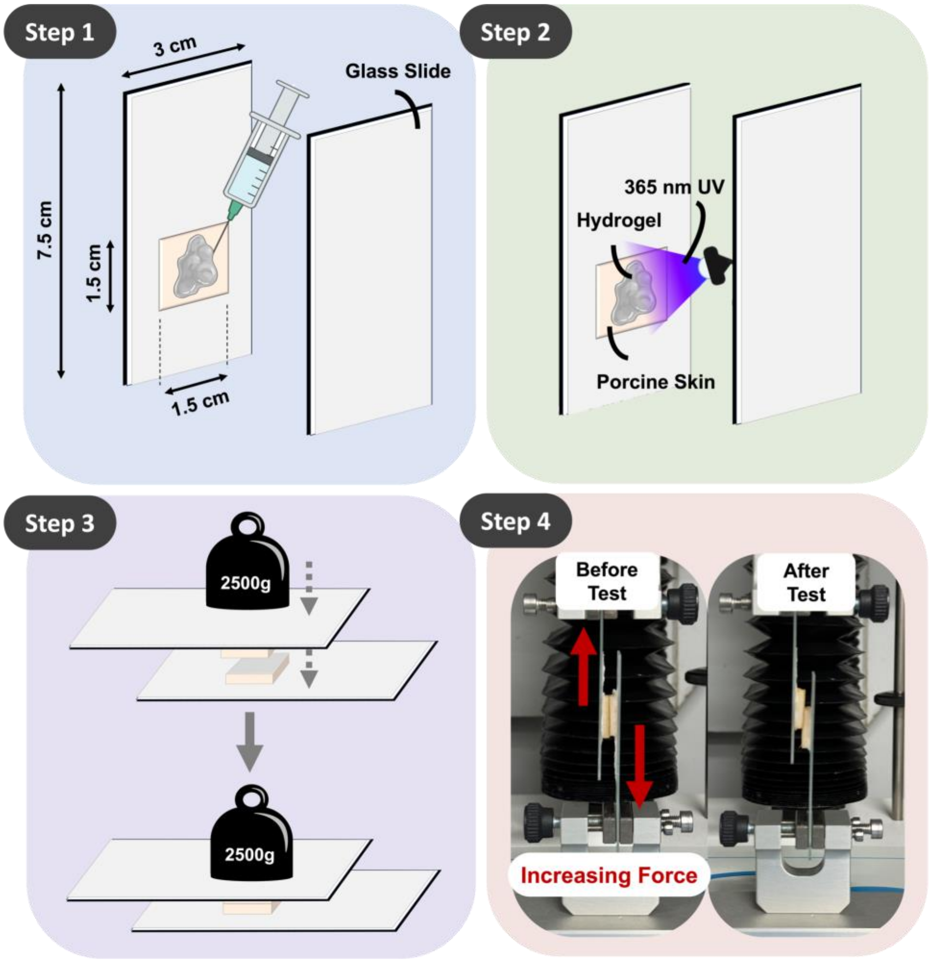

Figure S7. Scheme of the steps for two lap-shear adhesion measurement to test the hydrogel–tissue binding strength.

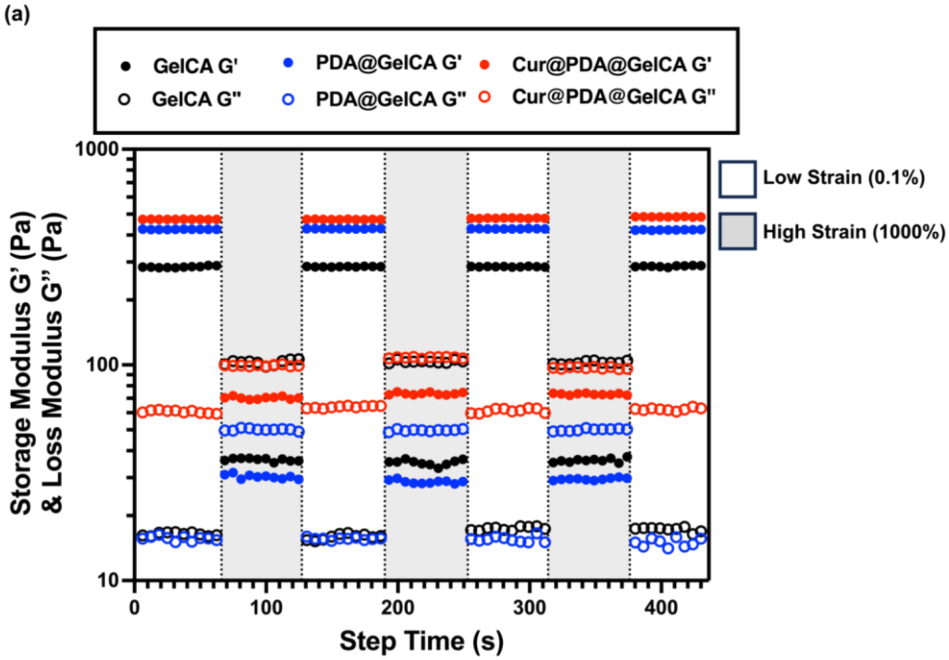

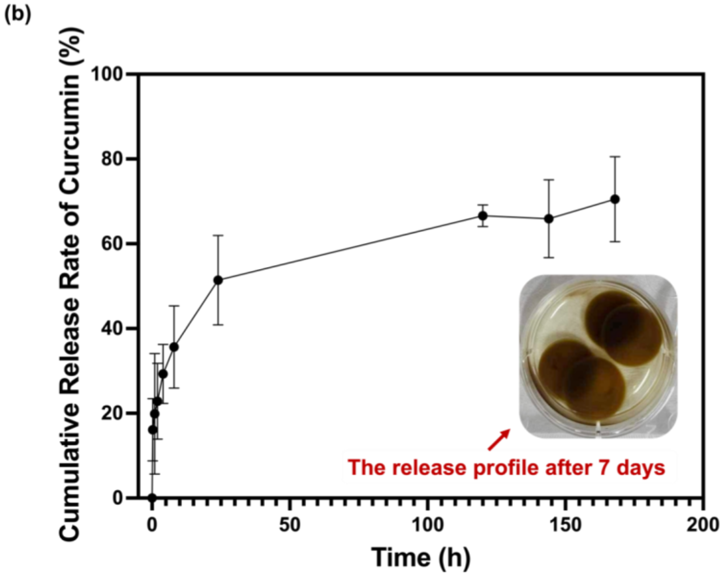

Figure S8. (a) The self-healing test conducted by continuous step strain sweep under low strain (0.1% strain) and high strain (1000% strain) at 1 rad/s with an 20 mm-diameter plate geometry (0 °angle) at 37 °C for 60 s. (b) Release profile of curcumin from Cur@PDA@GelCA hydrogel (2 mL Cur@PDA@GelCA Hydrogel in 5 mL PBS), the data were presented as mean ± SD (n = 3).

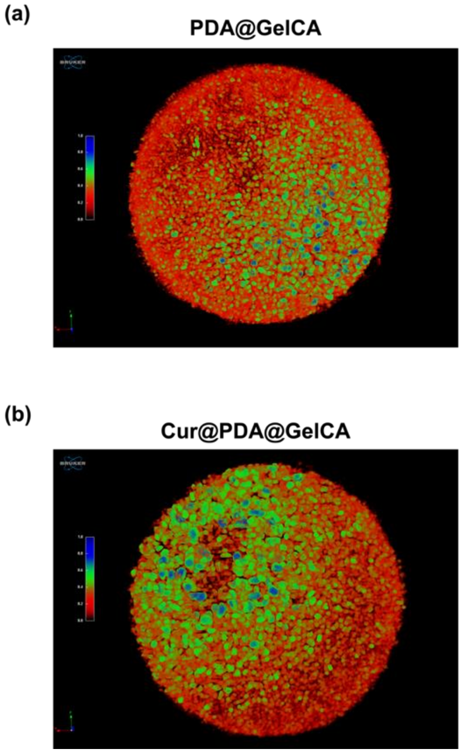

Figure S9. (a) Micro-CT image of PDA@GelCA and (b) Cur@PDA@GelCA hydrogel.

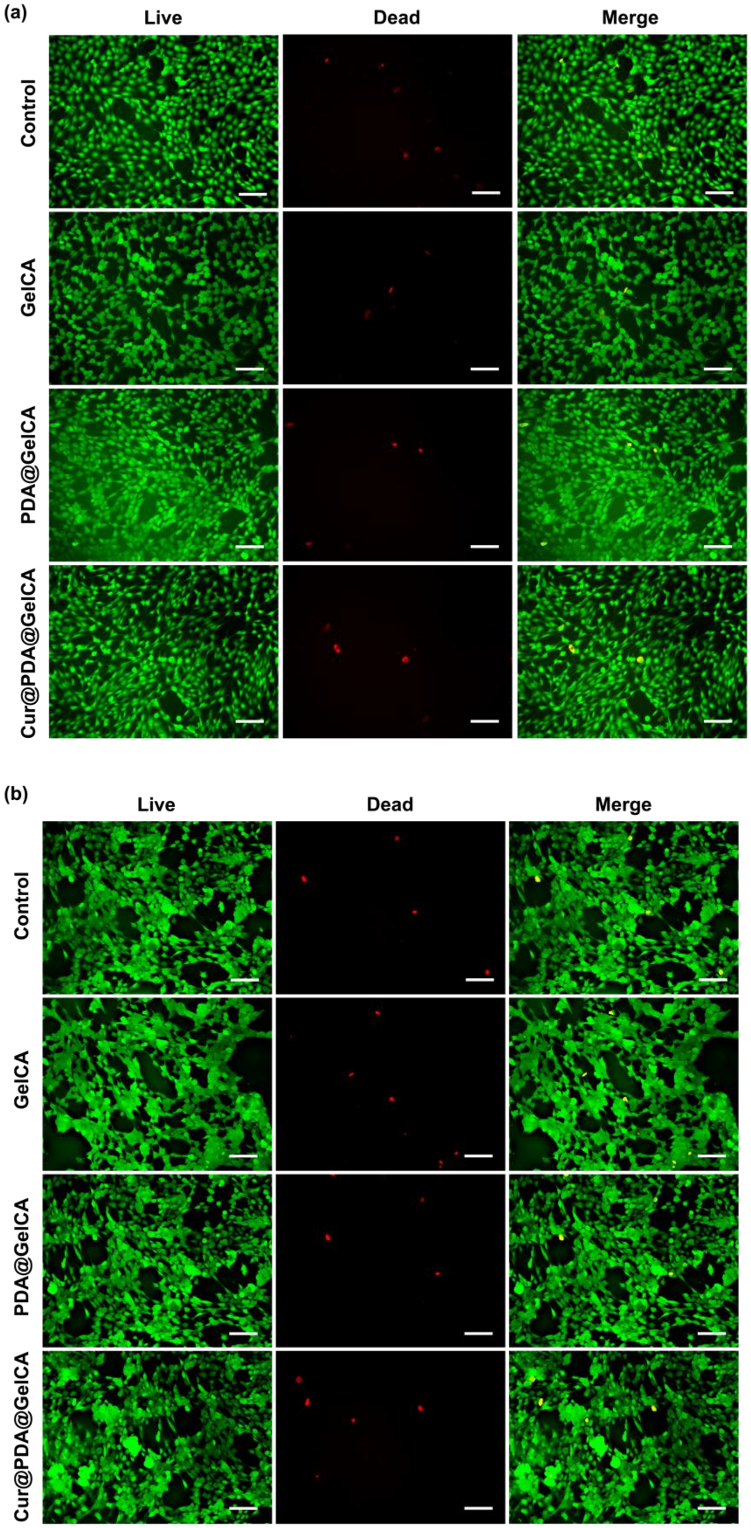

Figure S10. (a) Fluorescent staining of live and dead cells in RGC-5 (scale bars: 100 μm). (b) Fluorescent staining of live and dead cells in R661W (scale bars: 100 μm).
